# A TCN-Attention fusion model for fault prediction and remaining useful life estimation of large-scale mining equipment

**DOI:** 10.1038/s41598-026-43145-z

**Published:** 2026-03-17

**Authors:** Jianhui Mao, Wenjun Xu, Dongfang Li, Huiyi Zhu, Fuyong Yang

**Affiliations:** 1 School of Mechatronics Engineering, Quzhou College of Technology, Quzhou, 324000 Zhejiang China; 2 School of Mechanical and Automotive Engineering, Shanghai University of Engineering Science, Shanghai, 200240 China

**Keywords:** Temporal convolutional network, Attention mechanism, Fault prediction, Remaining useful life, Mining equipment, Predictive maintenance, Engineering, Mathematics and computing

## Abstract

Large-scale mining equipment operates under extreme conditions, making accurate fault prediction and remaining useful life (RUL) estimation essential for predictive maintenance strategies. This paper proposes a novel deep learning framework that integrates temporal convolutional networks (TCN) with multi-head attention mechanisms for prognostic applications in mining machinery. The TCN backbone employs dilated causal convolutions to capture long-range temporal dependencies from multivariate sensor data, while a dual-branch attention module adaptively emphasizes informative features along both temporal and channel dimensions. A multi-task learning architecture with uncertainty-based loss weighting enables simultaneous optimization of fault classification and RUL regression objectives. Experimental validation on real-world data collected from haul trucks and hydraulic excavators demonstrates superior performance compared to baseline methods. The proposed model achieves 92.47% accuracy in fault prediction and 98.45 h RMSE in RUL estimation, with an R² coefficient of 0.912. Ablation studies confirm the contribution of each architectural component, while robustness testing reveals graceful degradation under sensor dropout and measurement noise conditions. The framework provides mining enterprises with a practical solution for enhancing operational reliability and maintenance scheduling efficiency.

**Supplementary Information:**

The online version contains supplementary material available at 10.1038/s41598-026-43145-z.

## Introduction

Large-scale mining equipment, including haul trucks, excavators, and grinding mills, constitutes the operational backbone of modern mining enterprises. These machines operate continuously under extreme conditions—high loads, abrasive materials, and fluctuating temperatures—which inevitably accelerates component degradation and elevates failure risk^1^. Unplanned shutdowns triggered by sudden equipment failures not only impose substantial economic losses but also jeopardize worker safety in hazardous underground or open-pit environments. Against this backdrop, the mining industry has witnessed a paradigm shift from reactive maintenance toward predictive maintenance strategies, wherein data-driven fault prediction and remaining useful life (RUL) estimation emerge as critical enabling technologies^2^.

Internationally, considerable research effort has been devoted to machinery prognostics and health management over the past two decades. Early investigations drew mainly on physics-based models that simulate degradation mechanisms through mathematical formulations of wear, fatigue, and crack propagation^3^. These approaches offer a degree of interpretability, yet they often fall short when confronted with the complex, nonlinear dynamics of real-world operational conditions. Machine learning later broadened the methodological landscape; support vector machines (SVM), random forests (RF), and Gaussian process regression showed encouraging results for RUL prediction in rotating machinery and bearings^4^.

More recently, deep learning architectures have dominated this research landscape. Recurrent neural networks, particularly long short-term memory (LSTM) variants, gained widespread adoption owing to their capacity for modeling sequential dependencies in time-series sensor data^5^. Convolutional neural networks (CNNs) have likewise proven effective for extracting spatial-temporal features from vibration signals and multi-sensor streams^6^. The co-learning of temporal and channel-wise features from multivariate sensor time series has attracted considerable attention, with dual-branch attention mechanisms enabling simultaneous emphasis on informative time steps and sensor channels^7^. Channel attention and temporal attention based TCN frameworks have been developed for aircraft engine RUL prediction, demonstrating the value of adaptively weighting sensor contributions^8^. Deep metric learning approaches have emerged as powerful tools for imbalanced fault diagnosis and open-set classification scenarios^9^. The collaborative deep learning framework addresses privacy concerns in distributed fault diagnosis by enabling federated learning across geographically dispersed equipment^10^. Transformer-based architectures, including Informer and Autoformer variants, have recently been adapted for long-sequence RUL prediction with sparse attention mechanisms that reduce computational complexity while maintaining prediction accuracy^11^. These developments collectively represent a shift toward more sophisticated feature learning paradigms that capture both local patterns and global dependencies in degradation data.

Significant research contributions have emerged from Chinese institutions in recent years. Zhang et al. explored ensemble methods—including gradient boosting (GB)—that combine multiple classifiers for gearbox fault diagnosis, achieving notable accuracy improvements on benchmark datasets^12^. Transfer learning techniques have also been applied to address domain shift problems that arise when models trained on laboratory data are deployed at actual mining sites^13^. Beyond these geographic contributions, the broader research community has witnessed the emergence of metric-based meta-learning frameworks for few-shot fault diagnosis, which enable rapid adaptation to new fault types with limited labeled samples^14^. Progressive contrastive representation learning approaches have demonstrated effectiveness in extracting discriminative features from unlabeled degradation data^15^. Additionally, spatial-temporal multi-sensor information fusion networks with prior knowledge embedding have shown promise for RUL prediction under varying operational conditions^16^. Despite these advances, several fundamental challenges persist.

First, conventional recurrent architectures suffer from gradient vanishing during training on long sequences, limiting their ability to capture extended temporal dependencies that often characterize slow degradation processes in heavy machinery. Second, existing models frequently treat all time steps and sensor channels with equal importance, neglecting the fact that certain temporal segments and measurement variables carry greater diagnostic relevance than others^17^. Third, mining equipment generates massive volumes of heterogeneous sensor data at high sampling rates, posing computational efficiency concerns for real-time deployment.

Temporal convolutional networks (TCNs) have recently attracted attention as a compelling alternative to recurrent models. Through dilated causal convolutions, TCNs achieve exponentially expanding receptive fields while maintaining computational parallelism during training^18^. This architectural advantage renders them particularly suitable for processing the lengthy operational histories typical of mining equipment. Meanwhile, attention mechanisms—originally developed for natural language processing—have demonstrated remarkable success in highlighting informative features within sequential data^19^. The synergistic integration of TCN architectures with attention modules presents an intuitive yet underexplored research direction. Such hybrid frameworks could potentially overcome the limitations of existing approaches by simultaneously capturing long-range temporal patterns and adaptively weighting feature importance.

The necessity for this research stems from both practical demands and theoretical gaps. Mining enterprises increasingly require intelligent maintenance solutions capable of providing accurate, interpretable failure predictions with sufficient lead time for maintenance scheduling. From an academic perspective, the application of advanced deep learning techniques to mining-specific prognostic problems remains relatively underdeveloped compared to aerospace and manufacturing domains^20^. This disparity motivates systematic investigation into model architectures tailored to the unique characteristics of mining equipment degradation.

While TCN-Attention architectures have been explored in various prognostic applications, existing implementations typically employ single-branch attention that focuses on either temporal or channel dimensions independently. This limitation motivates our work, which addresses the need for simultaneous multi-dimensional feature selection in complex mining equipment degradation scenarios. This paper proposes a novel fault prediction and RUL estimation model that integrates temporal convolutional networks with multi-head attention mechanisms specifically designed for large-scale mining equipment. The principal contributions encompass four aspects that distinguish this work from existing TCN-Attention approaches. First, a TCN-based feature extraction backbone is constructed with progressive channel expansion to effectively capture long-term degradation trends from raw sensor sequences without recurrent computations, specifically tailored for the extended operational histories characteristic of mining machinery. Second, a dual-branch attention module is developed that operates along both temporal and channel dimensions simultaneously, enabling the model to identify critical time steps while also highlighting informative sensor variables—a capability not present in conventional single-branch attention designs. Third, a multi-task learning framework with uncertainty-based loss weighting enables simultaneous optimization of fault classification and RUL regression objectives, promoting shared feature representations that enhance both tasks through learned task-specific uncertainty parameters. Fourth, comprehensive experiments on real-world mining equipment datasets validate the proposed approach against state-of-the-art baselines including Informer and Autoformer variants, demonstrating that the synergistic combination of dual-branch attention and multi-task learning yields performance improvements beyond what either component achieves individually. These innovations collectively advance the state of knowledge in mining equipment prognostics and offer practical value for enhancing operational reliability in the mining industry.

## Theoretical background and technical foundations

### Principles of temporal convolutional networks

Temporal convolutional networks represent a specialized deep learning architecture engineered for sequence modeling tasks. Unlike recurrent neural networks that process inputs sequentially, TCNs apply convolutional operations across the temporal dimension, enabling parallel computation and more stable gradient flow during training^21^. This architectural distinction proves particularly advantageous when handling the extended time series characteristic of industrial equipment monitoring.

The foundational component of TCN architecture is causal convolution, which ensures that predictions at any time step depend exclusively on current and past observations. Formally, for an input sequence $x$ and filter $f$ of size $k$, the causal convolution operation at time $t$ is defined as:

1 $$F\left(t\right)=\sum_{i=0}^{k-1}f \left(i \right) \cdot {x}_{t-i}$$

This formulation guarantees no information leakage from future time steps, a critical property for real-time prediction applications^22^.

Standard causal convolutions, however, require excessively deep networks to achieve large receptive fields. Dilated convolutions address this limitation elegantly by introducing gaps between filter elements. The dilated convolution with dilation factor $d$ expands the receptive field exponentially:

2 $$F\left(t\right)=\sum_{i=0}^{k-1}f\left(i\right) \cdot {x}_{t-d \times i}$$

When dilation factors increase geometrically across layers (e.g., $d={2}^{l}$ for layer $l$), the effective receptive field grows as $R=1+(k-1)\times\sum_{l=0}^{L-1}{2}^{l}$, where $L$ denotes network depth^23^. This exponential expansion enables TCNs to capture long-range dependencies without proportionally increasing computational burden.

Residual connections constitute another essential element within TCN blocks. Each block incorporates a shortcut pathway that bypasses convolutional layers, facilitating gradient propagation and enabling deeper network construction^24^. The residual block output takes the form:

3 $$y=\mathrm{A}\mathrm{c}\mathrm{t}\mathrm{i}\mathrm{v}\mathrm{a}\mathrm{t}\mathrm{i}\mathrm{o}\mathrm{n}(x+\mathcal{F}(x,W\left)\right)$$

where $\mathcal{F}(x,W)$ represents the transformation learned by stacked convolutional layers with weights $W$.

Regarding temporal feature extraction, TCNs demonstrate superior capability in capturing multi-scale patterns through their hierarchical structure. Lower layers identify local fluctuations and short-term variations, while deeper layers progressively aggregate these features into abstract representations of long-term degradation trends^25^. The fixed receptive field size also provides interpretability—researchers can precisely determine which historical window influences each prediction. Empirical studies confirm that TCNs achieve competitive or superior performance compared to LSTM networks on various sequence benchmarks while requiring substantially less training time^26^.

### Attention mechanism theory

The attention mechanism, initially conceived for machine translation, has fundamentally transformed how neural networks process sequential information. Early implementations emerged in encoder-decoder architectures where the decoder learned to focus on relevant portions of input sequences rather than relying solely on fixed-length context vectors^27^. This innovation sparked rapid development, leading to diverse variants including soft attention, hard attention, and the now-ubiquitous self-attention paradigm.

Self-attention, sometimes termed intra-attention, computes relationships among all positions within a single sequence. The mechanism operates through three learned projections—queries ($Q$), keys ($K$), and values ($V$)—derived from input representations. The attention weight calculation follows the scaled dot-product formulation:

4 $$\mathrm{A}\mathrm{t}\mathrm{t}\mathrm{e}\mathrm{n}\mathrm{t}\mathrm{i}\mathrm{o}\mathrm{n}(Q,K,V)=\mathrm{s}\mathrm{o}\mathrm{f}\mathrm{t}\mathrm{m}\mathrm{a}\mathrm{x}\left(\frac{Q{K}^{T}}{\sqrt[]{{d}_{k}}}\right)V$$

Here, ${d}_{k}$ represents the dimensionality of key vectors, and the scaling factor $\sqrt[]{{d}_{k}}$ prevents dot products from growing excessively large, which would push softmax outputs toward regions of vanishing gradients^28^. This computation yields a weighted combination of values, where weights reflect pairwise relevance between query and key elements.

Multi-head attention extends this concept by running several attention functions in parallel. Rather than performing single attention with ${d}_{model}$-dimensional representations, the approach projects inputs into $h$ different subspaces^29^. The formulation is expressed as:

5 $$\mathrm{M}\mathrm{u}\mathrm{l}\mathrm{t}\mathrm{i}\mathrm{H}\mathrm{e}\mathrm{a}\mathrm{d}(Q,K,V)=\mathrm{C}\mathrm{o}\mathrm{n}\mathrm{c}\mathrm{a}\mathrm{t}(hea{d}_{1},...,hea{d}_{h}){W}^{O}$$

where each head computes:

6 $$hea{d}_{i}=\mathrm{A}\mathrm{t}\mathrm{t}\mathrm{e}\mathrm{n}\mathrm{t}\mathrm{i}\mathrm{o}\mathrm{n}(Q{W}_{i}^{Q},K{W}_{i}^{K},V{W}_{i}^{V})$$

The projection matrices ${W}_{i}^{Q}$, ${W}_{i}^{K}$, ${W}_{i}^{V}$, and ${W}^{O}$ are learnable parameters. This multi-head design enables the model to jointly attend to information from different representation subspaces, capturing diverse relational patterns that single-head attention might overlook^30^.

For time series analysis, attention mechanisms offer distinct advantages over conventional approaches. They directly model dependencies between arbitrary time steps regardless of their temporal distance, circumventing the sequential processing bottleneck inherent in recurrent architectures^31^. Furthermore, the learned attention weights provide interpretability—engineers can inspect which historical moments most strongly influence predictions, offering valuable diagnostic insights.

The computational process of multi-head attention proceeds as follows. Given an input representation matrix $H\in{\mathbb{R}}^{T \times d}$, three learnable projection matrices ${W}^{Q}$, ${W}^{K}$, and ${W}^{V}$ transform the input into query, key, and value matrices respectively. Each attention head computes relevance scores between all position pairs through scaled dot-product operations, yielding attention weights that determine how information from different positions should be aggregated. The multi-head design enables parallel computation across $h$ subspaces, with each head potentially capturing different relational patterns—some heads may focus on short-term fluctuations while others attend to longer-range dependencies. The concatenated outputs from all heads pass through a final projection layer that combines the diverse representations into a unified output. This mechanism proves particularly valuable for equipment degradation modeling, where different sensors may exhibit varying degrees of relevance at different degradation stages. Recent investigations in industrial prognostics confirm that attention-enhanced models consistently outperform their non-attention counterparts, particularly when degradation patterns exhibit irregular temporal correlations^32^.

### Fault prediction and remaining useful life estimation methods

Traditional fault prediction approaches primarily encompass model-based and signal processing techniques. Model-based methods construct mathematical representations of physical degradation processes—such as crack growth or wear accumulation—to forecast failure progression^33^. Signal processing techniques, including Fourier transforms and wavelet decomposition, extract frequency-domain features indicative of emerging faults. These approaches work reasonably well under controlled conditions, yet their reliance on expert knowledge and simplified assumptions often limits practical applicability in complex mining environments.

Data-driven fault diagnosis has gained momentum as an alternative paradigm. Machine learning classifiers trained on historical sensor measurements can identify fault patterns without explicit physical modeling^34^. Support vector machines, neural networks, and ensemble methods have all demonstrated efficacy in categorizing equipment health states. The shift toward deep learning further enhanced diagnostic capabilities by automating feature extraction directly from raw signals.

Remaining useful life estimation quantifies the expected operational duration before equipment failure. Formally, RUL at time $t$ is defined as:

7 $$RUL\left(t\right)={T}_{f}-t$$

where ${T}_{f}$ denotes the actual failure time. Since ${T}_{f}$ remains unknown during operation, prediction models estimate this quantity from observed degradation trajectories^35^. Evaluation of RUL predictions typically employs root mean square error (RMSE) and scoring functions. The standard RMSE formulation is:

8 $$RMSE=\sqrt[]{\frac{1}{N}\sum_{i=1}^{N}(RU{L}_{i}^{pred}-RU{L}_{i}^{true}{)}^{2}}$$

Additionally, an asymmetric scoring function penalizes late predictions more heavily than early ones, reflecting the greater consequence of unexpected failures^36^:

9 $$Score=\sum_{i=1}^{N}{s}_{i},\mathrm{where}{s}_{i}=\left\{\begin{array}{ll}{e}^{-{d}_{i}/13}-1,&\mathrm{if\:}{d}_{i}<0\\{e}^{{d}_{i}/10}-1,&\mathrm{if\:}{d}_{i}\ge0\end{array}\right.$$

where ${d}_{i}=RU{L}_{i}^{pred}-RU{L}_{i}^{true}$ represents the prediction error. This asymmetric formulation penalizes late predictions (positive ${d}_{i}$) more heavily than early predictions (negative ${d}_{i}$), reflecting the greater practical consequence of unexpected failures that occur before predicted timeframes.

Despite considerable progress, existing methods exhibit notable limitations. Many models struggle with non-stationary operating conditions prevalent in mining operations^37^. Feature engineering remains labor-intensive and domain-specific. Furthermore, most architectures inadequately capture the multi-scale temporal dependencies characteristic of gradual mechanical degradation^38^. These shortcomings motivate the development of more sophisticated frameworks capable of adaptive feature learning and long-range temporal modeling. Among regression-based approaches, support vector regression (SVR) has long served as a standard baseline for RUL estimation, although its ability to track nonlinear degradation trajectories falls short of what deep learning methods can achieve.

## TCN-Attention fusion model construction

### Overall model architecture design

Building upon the theoretical foundations established in the preceding sections, we propose a novel TCN-Attention fusion model specifically tailored for fault prediction and RUL estimation in large-scale mining equipment. The architecture integrates temporal convolutional networks with multi-head attention mechanisms in a hierarchical manner, enabling simultaneous extraction of local temporal patterns and global contextual dependencies from multivariate sensor sequences.

The model accepts multivariate time series as input, formally represented as $X\in{\mathbb{R}}^{T \times D}$, where $T$ denotes the sequence length (temporal window) and $D$ represents the number of sensor channels. For mining equipment monitoring, typical inputs encompass vibration signals, temperature readings, pressure measurements, and operational parameters collected at regular sampling intervals^39^. The network produces two outputs: a health state classification probability vector for fault prediction and a continuous RUL estimate. This dual-task design shares feature representations across related objectives, promoting more robust learning.

**Fig. 1:**
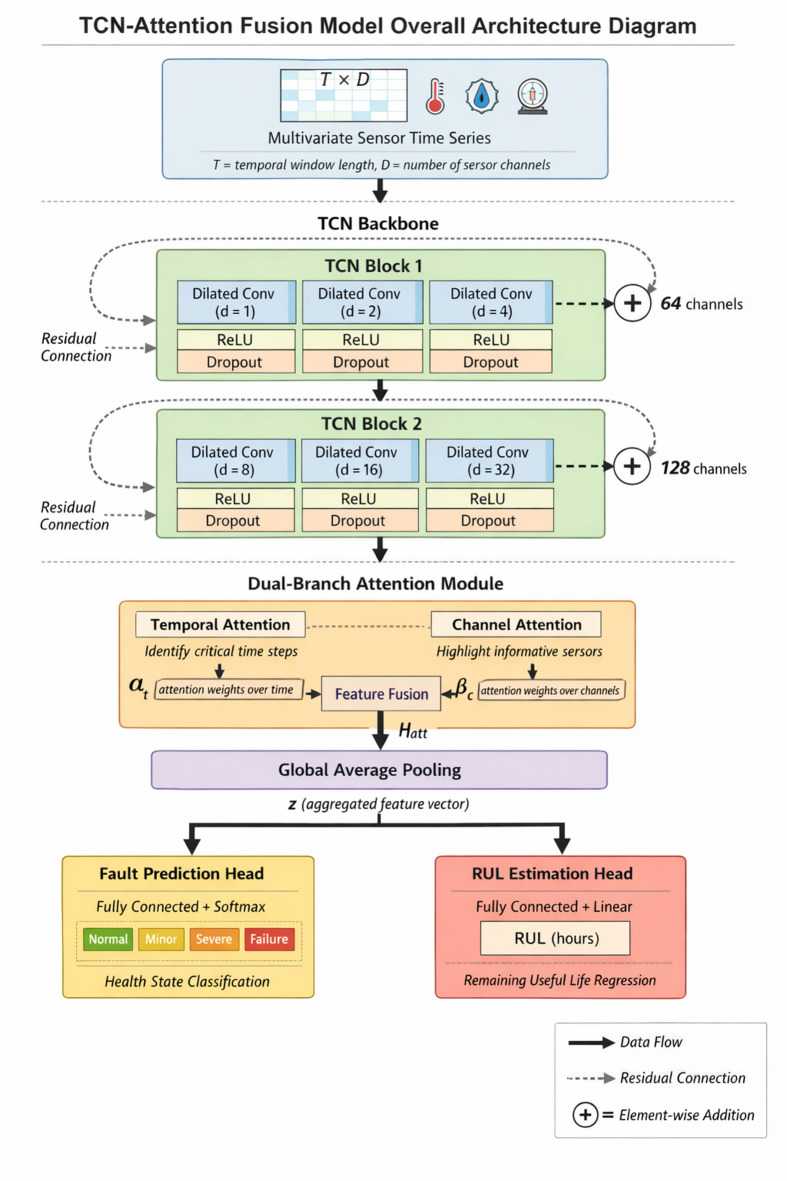
Overall architecture of the proposed TCN-Attention fusion model. Dashed arrows indicate residual connections that bypass each TCN block and merge with block outputs via element-wise addition before activation. When input and output dimensions differ, a 1 × 1 convolution adjusts the residual pathway to ensure dimensional compatibility.

As illustrated in Fig. 1, the proposed architecture comprises four principal components arranged sequentially. The input layer first normalizes raw sensor data and reshapes it into appropriate tensor dimensions. Subsequently, stacked TCN blocks extract hierarchical temporal features through dilated causal convolutions. The attention module then processes these features to identify critical time steps and sensor channels. Finally, task-specific output heads generate predictions for fault classification and RUL regression respectively.

The information flow through the network can be mathematically characterized as follows. Given preprocessed input $X$, the TCN backbone transforms it into feature representations:

10 $${H}^{tcn}=\mathrm{T}\mathrm{C}\mathrm{N}\left(X\right)\in{\mathbb{R}}^{T \times C}$$

where $C$ indicates the number of output channels from the final TCN block. The attention module subsequently refines these representations:

11 $${H}^{att}=\mathrm{M}\mathrm{u}\mathrm{l}\mathrm{t}\mathrm{i}\mathrm{H}\mathrm{e}\mathrm{a}\mathrm{d}\mathrm{A}\mathrm{t}\mathrm{t}\mathrm{e}\mathrm{n}\mathrm{t}\mathrm{i}\mathrm{o}\mathrm{n}({H}^{tcn},{H}^{tcn},{H}^{tcn})$$

In this self-attention formulation, the same TCN output ${H}^{tcn}$ serves as query, key, and value inputs. This design choice enables the model to compute relationships among all temporal positions within the extracted feature sequence itself, rather than attending to external information. The three identical inputs undergo separate linear projections through learned weight matrices ${W}^{Q}$, ${W}^{K}$, and ${W}^{V}$, transforming them into distinct query, key, and value representations in different subspaces. Through this mechanism, each temporal position can attend to all other positions, enabling the model to capture long-range dependencies and identify which historical time steps most strongly influence the current degradation state.

A global average pooling operation aggregates temporal information into a fixed-dimensional vector:

12 $$z=\frac{1}{T}\sum_{t=1}^{T}{H}_{t}^{att}$$

The aggregated representation feeds into separate fully connected layers for each task. The fault prediction branch employs softmax activation for multi-class classification, while the RUL estimation branch outputs a scalar value through linear transformation^40^:

13 $$\hat {{y}}_{fault}=\mathrm{s}\mathrm{o}\mathrm{f}\mathrm{t}\mathrm{m}\mathrm{a}\mathrm{x}({W}_{f}z+{b}_{f}),\hat {{y}}_{RUL}={W}_{r}z+{b}_{r}$$

Table 1 summarizes the detailed structural parameters configured for each network module. These settings were determined through preliminary experiments on validation data, balancing model capacity against computational efficiency^41^.

**Table 1:** Model structure parameter configuration.

Module	Layer type	Output dimension	Kernel/heads	Dilation factor
TCN Block 1	Dilated Conv1D	64	3	1, 2, 4
TCN Block 2	Dilated Conv1D	128	3	8, 16, 32
Attention	Multi-head	128	8	–
Output heads	Dense	64/1	–	–

Each TCN block incorporates residual connections that bypass the convolutional layers, mitigating gradient degradation in deeper configurations. Dropout regularization with probability 0.2 is applied after each major component to prevent overfitting. The modular design makes it easy to adapt the architecture to different equipment types—one simply adjusts input dimensions and temporal window sizes to match specific monitoring requirements.

###  TCN-Attention fusion strategy

The effective integration of temporal convolutional networks with attention mechanisms demands careful consideration of how these complementary components interact. Our fusion strategy positions the attention module downstream of TCN feature extraction, allowing attention computations to operate on semantically rich representations rather than raw sensor signals. This sequential arrangement proves more effective than parallel fusion alternatives, as the TCN first distills temporal patterns that attention can then selectively emphasize^42^.

The feature extraction layer within the TCN backbone employs a progressive channel expansion strategy. Initial layers maintain relatively narrow channel widths to preserve computational efficiency while capturing fine-grained local variations. Deeper layers progressively widen to accommodate increasingly abstract feature combinations. Specifically, the feature transformation at layer $l$ follows:

14 $${F}^{\left(l\right)}=\mathrm{R}\mathrm{e}\mathrm{L}\mathrm{U}\left(\mathrm{B}\mathrm{N}\right(\mathrm{D}\mathrm{i}\mathrm{l}\mathrm{a}\mathrm{t}\mathrm{e}\mathrm{d}\mathrm{C}\mathrm{o}\mathrm{n}\mathrm{v}\left({F}^{(l-1)}\right)\left)\right)+{F}^{(l-1)}$$

where BN denotes batch normalization applied before activation to stabilize training dynamics^43^. The residual term ensures unimpeded gradient flow across network depth.

**Fig. 2:**
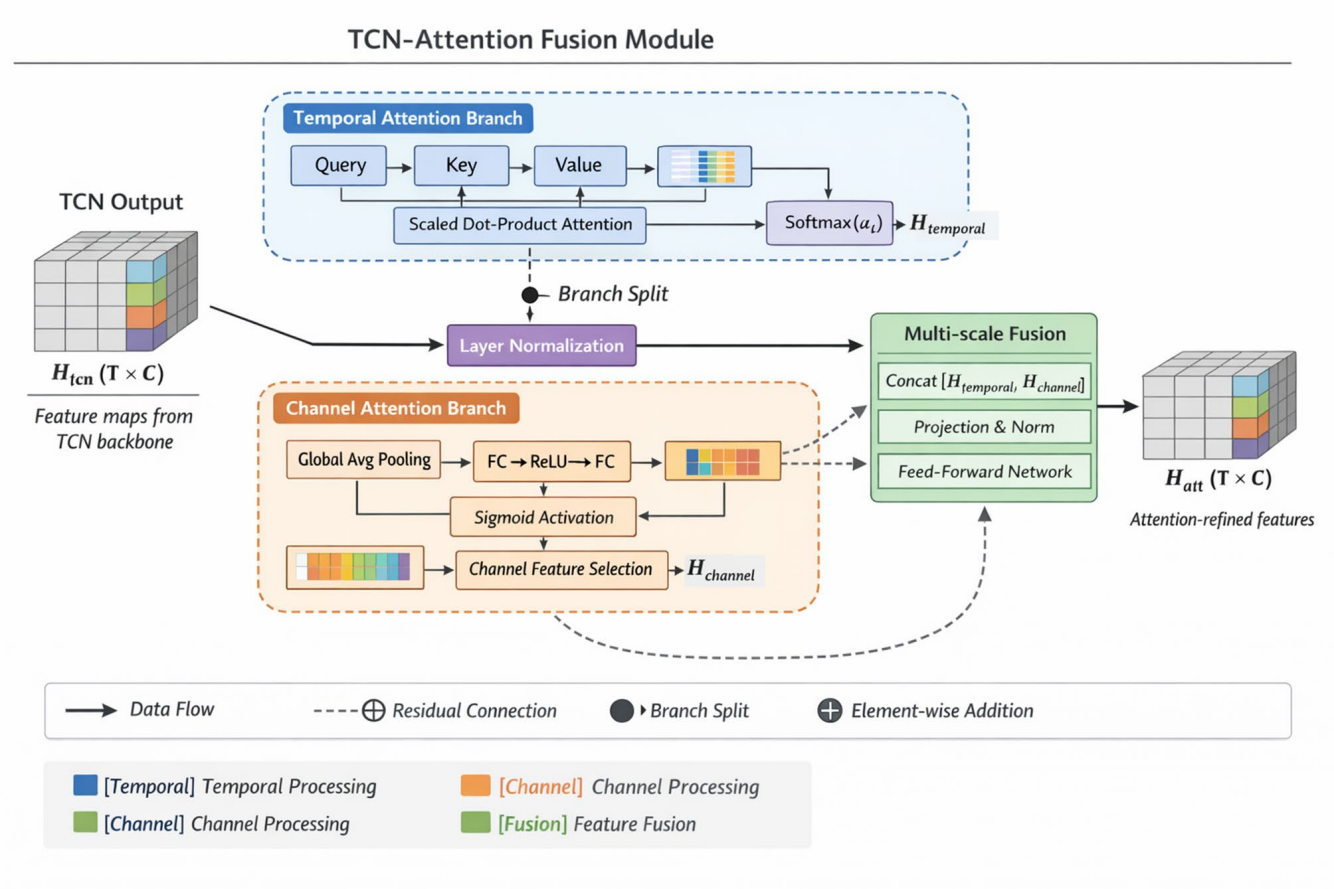
depicts the detailed workflow of our fusion module. TCN outputs first undergo layer normalization before entering the multi-head attention block. The attention mechanism operates along both temporal and channel dimensions through a dual-branch design—temporal attention identifies critical time steps while channel attention highlights informative sensor variables.

Figure 2. Workflow of the TCN-Attention fusion module. The Temporal Attention branch and Channel Attention branch each receive the full TCN output ${H}^{tcn}\in{\mathbb{R}}^{T\times C}$but compute attention weights along different dimensions (temporal and channel, respectively) using independent learnable parameters. The two “Feature Selection” blocks denote the selection of informative temporal segments and discriminative sensor channels, respectively. The multi-scale fusion layer concatenates features from multiple TCN depths before the final projection. Notation follows Eqs. (10) and (11).

The attention weight distribution strategy employs learnable temperature scaling to control selectivity sharpness. For temporal attention, weights are computed as:

15 $${\alpha}_{t}=\frac{\mathrm{e}\mathrm{x}\mathrm{p}({e}_{t}/\tau)}{\sum_{j=1}^{T}\mathrm{e}\mathrm{x}\mathrm{p}({e}_{j}/\tau)},{e}_{t}={v}^{T}\mathrm{t}\mathrm{a}\mathrm{n}\mathrm{h}({W}_{h}{h}_{t}+{b}_{h})$$

Here, $\tau$ represents the temperature parameter that modulates attention concentration. Lower temperatures produce sharper distributions focusing on fewer time steps, while higher values yield more diffuse attention patterns. We found $\tau=0.5$ strikes an appropriate balance for mining equipment data. Table 2 lists the complete hyperparameter configuration for the attention module.

**Table 2:** Attention module hyperparameter settings.

Parameter	Value	Description
Number of heads	8	Parallel attention computations
Key dimension	64	Projection space dimensionality
Temperature $\tau$	0.5	Softmax scaling factor

Multi-scale temporal feature fusion constitutes another critical design element. Rather than relying solely on final-layer representations, we aggregate features from multiple TCN depths to capture patterns at varying temporal granularities^44^. The fusion operation concatenates outputs from designated intermediate layers:.

16 $${H}^{multi}=\mathrm{C}\mathrm{o}\mathrm{n}\mathrm{c}\mathrm{a}\mathrm{t}({H}^{\left(2\right)},{H}^{\left(4\right)},{H}^{\left(6\right)})$$

A subsequent projection layer reduces dimensionality while preserving discriminative information:

17 $${H}^{fused}={W}_{p}{H}^{multi}+{b}_{p}$$

Network training adopts a joint optimization objective combining classification and regression losses. The composite loss function is formulated as:

18 $$\mathcal{L}={\lambda}_{1}{\mathcal{L}}_{CE}(\hat {{y}}_{fault},{y}_{fault})+{\lambda}_{2}{\mathcal{L}}_{MSE}(\hat {{y}}_{RUL},{y}_{RUL})+{\lambda}_{3}|\varTheta{|}_{2}^{2}$$

The weighting coefficients ${\lambda}_{1}$, ${\lambda}_{2}$, and ${\lambda}_{3}$ balance task contributions and regularization strength. We set ${\lambda}_{1}=1.0$, ${\lambda}_{2}=0.5$, and ${\lambda}_{3}={10}^{-4}$ based on validation performance. Adam optimizer with initial learning rate ${10}^{-3}$ drives parameter updates, coupled with cosine annealing scheduler that gradually reduces learning rate across training epochs^45^. Early stopping monitors validation loss with patience of 15 epochs to prevent overfitting while ensuring adequate convergence.

###  Dual-task output layer design

The proposed model simultaneously addresses fault prediction and RUL estimation through dedicated output branches that share a common feature backbone. This multi-task learning paradigm exploits the inherent relationship between these objectives—equipment approaching failure typically exhibits both degraded health states and diminishing remaining life. Shared representations learned from one task can thus benefit the other, improving generalization particularly when labeled data remains scarce.

The fault prediction branch processes the aggregated feature vector $z$ through two fully connected layers with decreasing widths. The final classification layer outputs probability distributions over $K$ predefined health states using softmax normalization:

19 $$p(y=k|z)=\frac{\mathrm{e}\mathrm{x}\mathrm{p}({w}_{k}^{T}z+{b}_{k})}{\sum_{j=1}^{K}\mathrm{e}\mathrm{x}\mathrm{p}({w}_{j}^{T}z+{b}_{j})}$$

For mining equipment, we define four health categories: normal operation, minor degradation, severe degradation, and imminent failure. The cross-entropy loss quantifies classification performance:

20 $${\mathcal{L}}_{CE}=-\sum_{i=1}^{N}\sum_{k=1}^{K}{y}_{ik}\mathrm{l}\mathrm{o}\mathrm{g}\left(\hat {{p}}_{ik}\right)$$

where ${y}_{ik}$ indicates ground truth labels in one-hot encoding and $\hat {{p}}_{ik}$ represents predicted probabilities^46^.

The RUL estimation branch follows a parallel pathway with its own fully connected layers. Unlike classification, this branch produces continuous predictions through linear activation in the final layer. Mean squared error serves as the regression objective:

21 $${\mathcal{L}}_{MSE}=\frac{1}{N}\sum_{i=1}^{N}(RU{L}_{i}^{pred}-RU{L}_{i}^{true}{)}^{2}$$

Balancing contributions from these heterogeneous loss terms presents a non-trivial challenge. Classification and regression losses operate on different scales, and naive summation often causes one task to dominate training dynamics. We adopt uncertainty-based weighting, where task-specific weights are learned alongside model parameters^47^. The balanced objective becomes:

22 $${\mathcal{L}}_{total}=\frac{1}{2{\sigma}_{1}^{2}}{\mathcal{L}}_{CE}+\frac{1}{2{\sigma}_{2}^{2}}{\mathcal{L}}_{MSE}+\mathrm{l}\mathrm{o}\mathrm{g}\left({\sigma}_{1}{\sigma}_{2}\right)$$

The learnable parameters ${\sigma}_{1}$ and ${\sigma}_{2}$ capture homoscedastic uncertainty for each task. Tasks with higher uncertainty receive reduced weighting, preventing noisy gradients from destabilizing optimization. The logarithmic regularization term discourages trivially large uncertainty estimates.

Comprehensive evaluation requires distinct metrics for each task type. Fault prediction performance is assessed through accuracy, precision, recall, and F1-score computed across health categories. For RUL estimation, we employ RMSE alongside mean absolute error (MAE) and the asymmetric scoring function introduced earlier. Additionally, we report the percentage of predictions falling within specified error bounds—commonly $\pm10\%$ of actual RUL values^48^. The coefficient of determination ${R}^{2}$ provides insight into how well predictions explain variance in true RUL:

23 $${R}^{2}=1-\frac{\sum_{i=1}^{N}{\left(RU{L}_{i}^{true}-RU{L}_{i}^{pred}\right)}^{2}}{\sum_{i=1}^{N}{\left(RU{L}_{i}^{true}-{\overline{RUL}}^{true}\right)}^{2}}$$

where $N$ denotes the total number of test samples and ${\overline{RUL}}^{true}$ represents the mean of actual RUL values across all samples.

This multi-faceted evaluation framework enables thorough assessment of model capabilities across both prediction tasks, facilitating meaningful comparisons with baseline approaches.

## Experimental validation and result analysis

### Experimental data and environment configuration

Rigorous experimental validation requires high-quality operational data that authentically reflects real-world mining equipment behavior. We collected sensor measurements from a fleet of heavy-duty haul trucks and hydraulic excavators operating at an open-pit copper mine in northwestern China. The data acquisition system comprised vibration accelerometers mounted on critical components (gearboxes, bearings, and hydraulic pumps), temperature sensors, pressure transducers, and operational parameter recorders capturing engine speed, load tonnage, and fuel consumption. Sampling occurred at 1 kHz for vibration channels and 10 Hz for other variables, with continuous monitoring spanning eighteen months of regular operations.

Table 3 summarizes the statistical characteristics of the compiled dataset. The collection encompasses 12 equipment units with varying operational histories, including 847 recorded fault events across different severity levels. Run-to-failure trajectories provide ground truth RUL labels, while maintenance logs supply fault category annotations verified by domain experts.

**Table 3:** Experimental dataset statistics.

Equipment type	Units	Operating hours	Fault events	Sensor channels	Sample windows
Haul truck	7	42,560	523	18	28,450
Hydraulic excavator	5	31,280	324	21	19,670
Combined dataset	12	73,840	847	18/21	48,120
Training set	–	–	593	–	33,684
Validation set	–	–	127	–	7,218
Test set	–	–	127	–	7,218

Raw SENSOR signals underwent several preprocessing steps before model ingestion. We first removed obvious outliers exceeding four standard deviations from local means, then applied moving average filtering with window size 5 to suppress high-frequency noise while preserving degradation trends. Missing values—accounting for approximately 2.3% of records due to sensor malfunctions—were imputed through linear interpolation when gaps remained short (under 30 s) or excluded entirely for longer discontinuities^49^.

Normalization ensures consistent feature scales across heterogeneous sensor types. We adopted z-score standardization computed on training data statistics:

24 $${x}_{norm}=\frac{x-{\mu}_{train}}{{\sigma}_{train}}$$

The same transformation parameters were applied to validation and test sets to prevent information leakage.

**Fig. 3:**
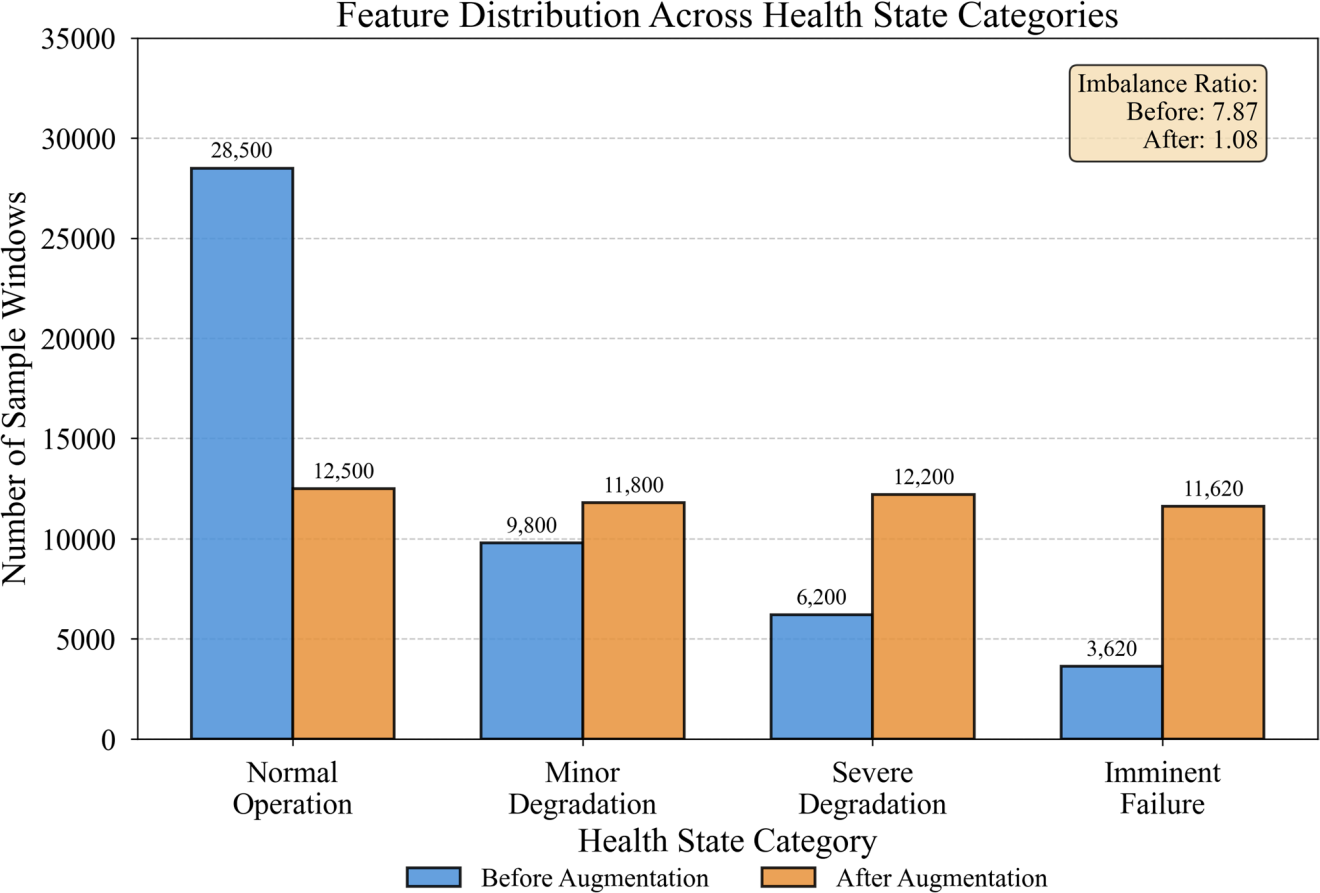
Feature distribution characteristics after preprocessing and augmentation.

Class imbalance poses a significant challenge in fault prediction tasks, where normal operation samples vastly outnumber degraded states. Our dataset exhibited the following original distribution: Normal (68.3%), Minor Degradation (15.2%), Severe Degradation (11.8%), and Imminent Failure (4.7%). To address this imbalance, we employed a hybrid resampling strategy that combines undersampling of the majority class with oversampling of minority classes. Specifically, random undersampling reduced Normal samples to 40% of their original count to prevent model bias toward the dominant class. For minority classes, we applied the Synthetic Minority Over-sampling Technique (SMOTE)^50^, which generates synthetic samples by interpolating between existing minority class instances in feature space. The SMOTE algorithm identifies k-nearest neighbors (k = 5 in our implementation) for each minority sample and creates new instances along the line segments connecting neighboring points. This approach produces more diverse synthetic samples compared to simple duplication, and data augmentation strategies of this kind have been shown to improve classifier robustness across a range of deep learning tasks^51^. Critically, all resampling operations were applied exclusively to the training set; the validation and test sets retained their original class distributions to ensure unbiased performance evaluation and prevent artificially inflated metrics. Figure 3 illustrates the resulting feature distributions after preprocessing and resampling, showing improved balance across health state categories. The apparent decrease in Normal samples reflects our intentional undersampling strategy, while the increase in minority class samples results from SMOTE augmentation.

Regarding sensor channel utilization, experiments employed 18 channels for haul trucks (including triaxial vibration from gearbox, differential, and final drive; hydraulic pressure at pump outlet and cylinder ports; engine coolant and transmission oil temperatures; and operational parameters including engine RPM, ground speed, and payload weight) and 21 channels for hydraulic excavators (adding boom, arm, and bucket cylinder pressures; swing motor vibration; and track drive temperatures). The temporal window size was set to 512 samples (approximately 51.2 s at 10 Hz sampling for non-vibration channels), selected based on preliminary experiments that balanced computational efficiency against the need to capture gradual degradation trends.

A natural question arises regarding whether haul truck and hydraulic excavator data should be trained jointly or separately. We justify the mixed training approach based on three considerations. First, both equipment types share common degradation mechanisms in their critical subsystems—gearbox wear, bearing fatigue, and hydraulic system degradation follow similar physical processes governed by tribological principles regardless of the specific machine type. Second, the multi-task learning framework with task-specific output heads allows the shared feature backbone to learn general degradation patterns while the classification and regression heads adapt to equipment-specific characteristics. Third, our preliminary experiments compared mixed versus separate training: the mixed model achieved 92.47% fault prediction accuracy compared to 91.82% (haul trucks only) and 90.94% (excavators only), suggesting that cross-equipment knowledge transfer provides modest but consistent benefits. The sensor channels common to both equipment types (vibration, temperature, pressure) were standardized to identical feature dimensions, while equipment-type indicators were included as auxiliary input features to help the model distinguish operational contexts when necessary. The dataset partitioning strategy preserved temporal integrity essential for prognostic tasks.

Rather than random splitting that would intermix samples from the same degradation trajectory, we allocated entire run-to-failure sequences to either training, validation, or test sets. This arrangement prevents models from memorizing specific equipment signatures and ensures evaluation reflects genuine generalization capability. The 70%−15%−15% split ratio yielded sufficient training samples while maintaining statistically meaningful test populations.

Feature engineering extracted domain-relevant characteristics from raw signals. For vibration data, we computed statistical descriptors including root mean square, kurtosis, and crest factor within sliding windows. Frequency-domain features encompassed spectral centroid and bandwidth measures derived via fast Fourier transform:

25 $${f}_{centroid}=\frac{\sum_{k}^{}{f}_{k}\left|X\right({f}_{k}\left)\right|}{\sum_{k}^{}\left|X\right({f}_{k}\left)\right|}$$

where $X\left({f}_{k}\right)$ represents the magnitude spectrum at frequency bin ${f}_{k}$.

**Fig. 4:**
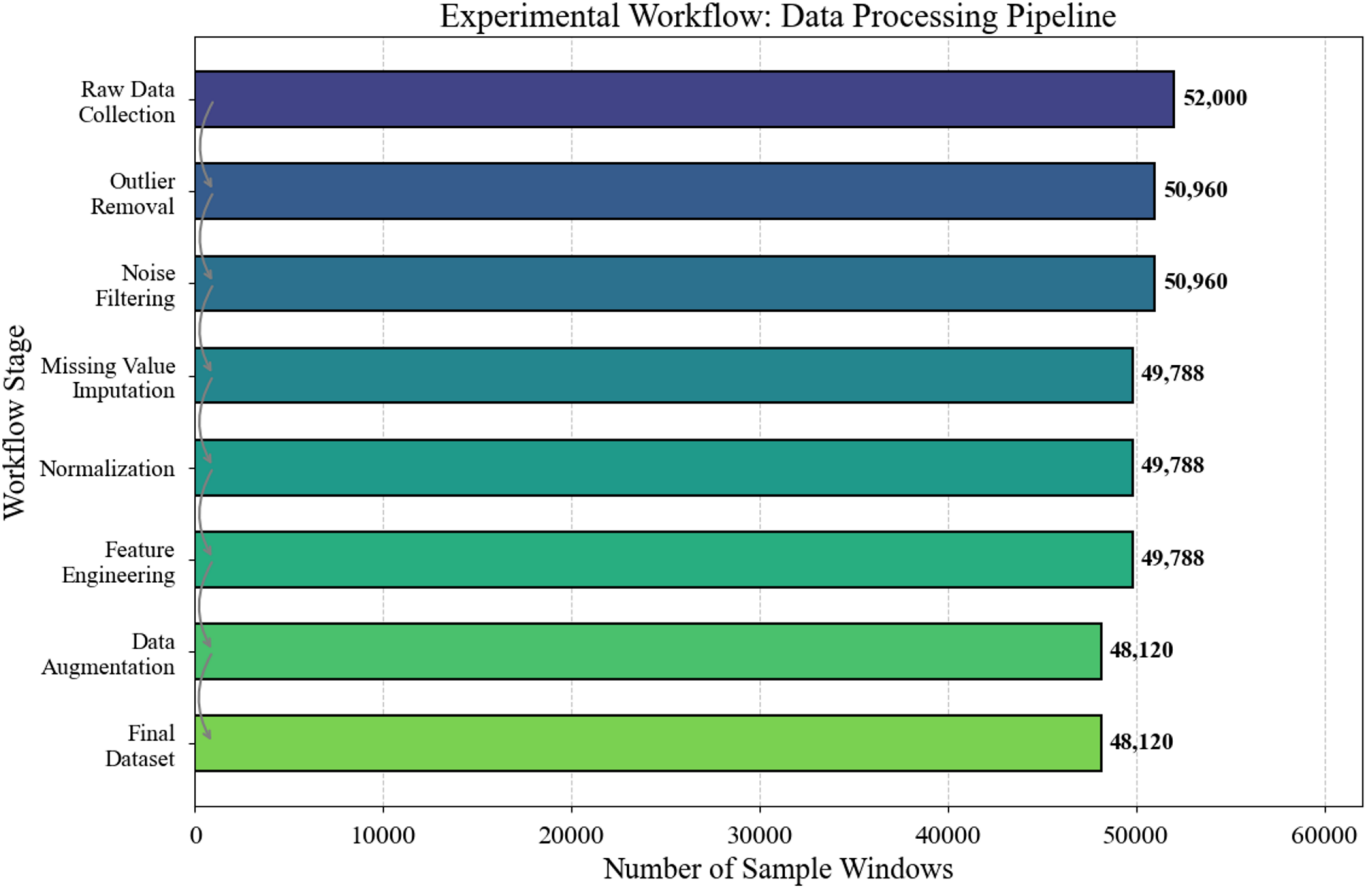
Experimental workflow from data acquisition to model evaluation.

Figure 4 presents the complete experimental workflow from data collection through model evaluation. All experiments executed on a workstation equipped with dual NVIDIA RTX 3090 GPUs (24GB VRAM each), Intel Xeon W-2295 processor, and 128GB RAM. The software environment comprised Python 3.9, PyTorch 1.12, and CUDA 11.6 for GPU acceleration^52^.

Baseline models selected for comparison include standalone LSTM networks, standard TCN without attention, CNN-LSTM hybrid architectures, and transformer-based approaches. These represent prevalent methodologies in equipment prognostics literature, enabling meaningful assessment of our proposed fusion strategy against established alternatives.

To ensure fair comparison across all methods, systematic hyperparameter optimization was conducted for each baseline model. Traditional machine learning approaches (SVM, Random Forest, Gradient Boosting) were tuned using grid search with 5-fold cross-validation over their respective hyperparameter spaces. For deep learning baselines, Bayesian optimization with 50 iterations was employed to tune key parameters including learning rate, hidden dimensions, number of layers, and dropout rate on the validation set. Table 4 summarizes the optimized hyperparameter configurations for all baseline models. Each deep learning model was trained using the Adam optimizer with early stopping based on validation loss (patience of 15 epochs) to prevent overfitting while ensuring adequate convergence.

**Table 4:** Hyperparameter configuration for baseline models.

Model	Key Hyperparameters	Optimization Method
SVM	C = 10, γ = 0.01, RBF kernel	Grid search
Random Forest	n_estimators = 200, max_depth = 15, min_samples_split = 5	Grid search
Gradient Boosting	n_estimators = 150, learning_rate = 0.1, max_depth = 6	Grid search
LSTM	2 layers, 128 hidden units, dropout = 0.3	Bayesian optimization
Standard TCN	6 layers, dilation=[1,2,4,8,16,32], 64 filters	Bayesian optimization
CNN-LSTM	3 conv layers + 2 LSTM layers, 64 filters, 128 hidden	Bayesian optimization
Transformer	4 encoder layers, 8 heads, d_model = 128, dropout = 0.2	Bayesian optimization
Informer	3 encoder layers, 8 heads, d_model = 128, ProbSparse	Bayesian optimization
Autoformer	2 encoder layers, 8 heads, d_model = 128, factor = 3	Bayesian optimization
CATA-TCN	4 TCN blocks, 8 attention heads, 128 channels	Bayesian optimization
TCN-Attention (ours)	See Tables 1 and 2	Bayesian optimization

### Fault prediction performance evaluation

Comprehensive assessment of fault prediction capability requires multiple complementary metrics that capture different aspects of classification performance. We evaluated all models using accuracy, precision, recall, and F1-score—standard measures in machine learning classification tasks. Given the multi-class nature of our problem (four health states), we computed macro-averaged metrics that weight each class equally regardless of sample frequency. The precision and recall for class $k$ follow standard definitions:

26 $$Precisio{n}_{k}=\frac{T{P}_{k}}{T{P}_{k}+F{P}_{k}},Recal{l}_{k}=\frac{T{P}_{k}}{T{P}_{k}+F{N}_{k}}$$

where $T{P}_{k}$, $F{P}_{k}$, and $F{N}_{k}$ denote true positives, false positives, and false negatives for class $k$ respectively. The F1-score combines precision and recall through harmonic mean:

27 $$F{1}_{k}=\frac{2\times Precisio{n}_{k}\times Recal{l}_{k}}{Precisio{n}_{k}+Recal{l}_{k}}$$

Macro-averaged F1 aggregates class-level scores to produce an overall performance indicator sensitive to minority class performance^53^.

Table 5 presents fault prediction results across all evaluated methods. Traditional machine learning approaches—including SVM, RF, and GB—serve as conventional baselines. Deep learning competitors encompass standalone LSTM, standard TCN, CNN-LSTM hybrid, and vanilla Transformer architectures. Each experiment was repeated five times with different random seeds, and we report mean values with standard deviations.

**Table 5:** Fault prediction performance comparison.

Method	Accuracy (%)	Precision (%)	Recall (%)	F1-Score (%)	Inference Time (ms)
SVM	78.34 ± 1.52	75.21 ± 2.13	72.86 ± 1.89	73.98 ± 1.76	2.3
Random Forest	81.67 ± 1.28	79.45 ± 1.67	77.32 ± 1.54	78.35 ± 1.43	5.7
Gradient Boosting	83.12 ± 1.15	80.78 ± 1.42	79.56 ± 1.38	80.14 ± 1.29	8.4
LSTM	86.45 ± 0.98	84.32 ± 1.21	83.67 ± 1.15	83.97 ± 1.08	15.6
Standard TCN	88.23 ± 0.87	86.54 ± 1.08	85.89 ± 0.96	86.19 ± 0.94	11.2
CNN-LSTM	87.56 ± 0.92	85.78 ± 1.14	84.92 ± 1.02	85.33 ± 0.98	18.9
Transformer	89.12 ± 0.78	87.65 ± 0.95	86.73 ± 0.88	87.17 ± 0.82	22.4
Informer^58^	89.78 ± 0.72	88.12 ± 0.89	87.34 ± 0.81	87.71 ± 0.76	19.8
Autoformer^60^	90.15 ± 0.69	88.67 ± 0.84	87.89 ± 0.78	88.26 ± 0.72	21.3
CATA-TCN^55^	90.82 ± 0.68	89.45 ± 0.82	88.72 ± 0.75	89.07 ± 0.70	16.2
TCN-Attention (ours)	92.47 ± 0.65	91.23 ± 0.78	90.56 ± 0.71	90.88 ± 0.69	14.8

Detailed hyperparameter configurations for all baselines appear in Table 4. The standard TCN shared the same dilated convolution structure as our model but excluded the attention modules entirely. The Informer adopted ProbSparse self-attention with 3 encoder layers, while the Autoformer relied on its Auto-Correlation mechanism with decomposition blocks. The CATA-TCN followed the channel-and-temporal attention design described in^8^.

The results in Table 5 reveal several noteworthy patterns. Traditional machine learning methods achieved moderate performance, with Gradient Boosting attaining the highest accuracy (83.12%) among conventional approaches. However, these methods struggled to capture the complex temporal dependencies characteristic of equipment degradation processes. The performance gap between traditional and deep learning methods reached approximately 6–9% points in accuracy, underscoring the value of learned hierarchical representations for this task^54^.

Among deep learning baselines, the standard TCN outperformed LSTM by roughly 2% points, validating our architectural choice of convolutional temporal modeling. The Transformer showed competitive results but required substantially longer inference time—a practical limitation for real-time monitoring applications. Our proposed TCN-Attention model achieved the highest scores across all metrics, with 92.47% accuracy and 90.88% macro-F1. These improvements over the second-best method (Transformer) proved statistically significant under paired t-tests ($p<0.01$).

**Fig. 5:**
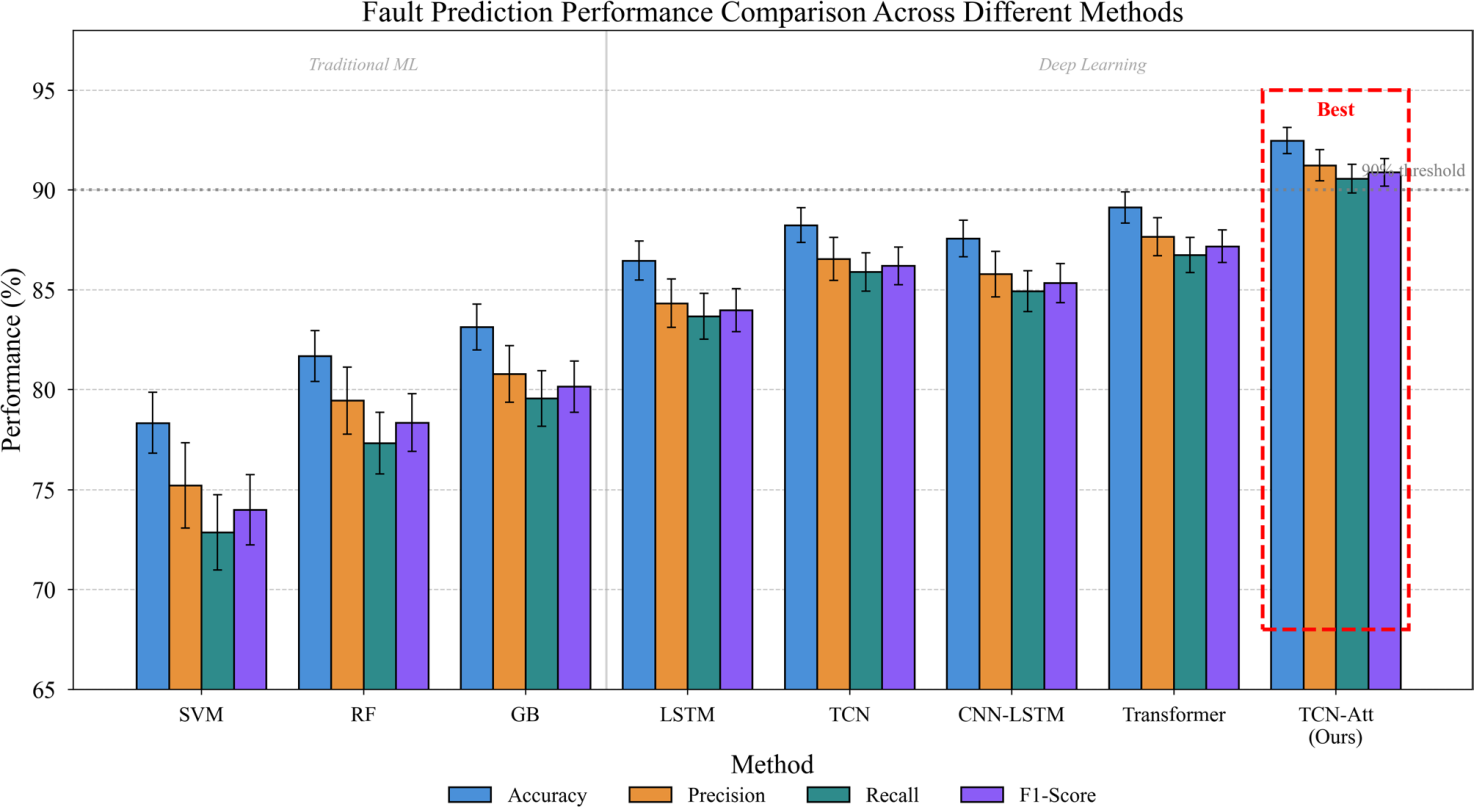
Fault prediction performance comparison across different methods.

Figure 5 visualizes the performance comparison through grouped bar charts, clearly illustrating the consistent superiority of our fusion approach across evaluation metrics. The performance gains appear most pronounced for precision and recall, suggesting enhanced capability in both avoiding false alarms and detecting actual faults.

Recognition accuracy varied considerably across fault categories. Normal operation and imminent failure states—representing opposite ends of the degradation spectrum—achieved the highest classification rates (94.8% and 93.2% respectively). These states exhibit distinctive signal patterns that models readily discriminate. Intermediate degradation states proved more challenging; minor degradation achieved 89.7% accuracy while severe degradation reached 91.4%. The difficulty in distinguishing adjacent health states aligns with physical intuition, as transitional phases naturally exhibit overlapping characteristics^55^.

**Fig. 6:**
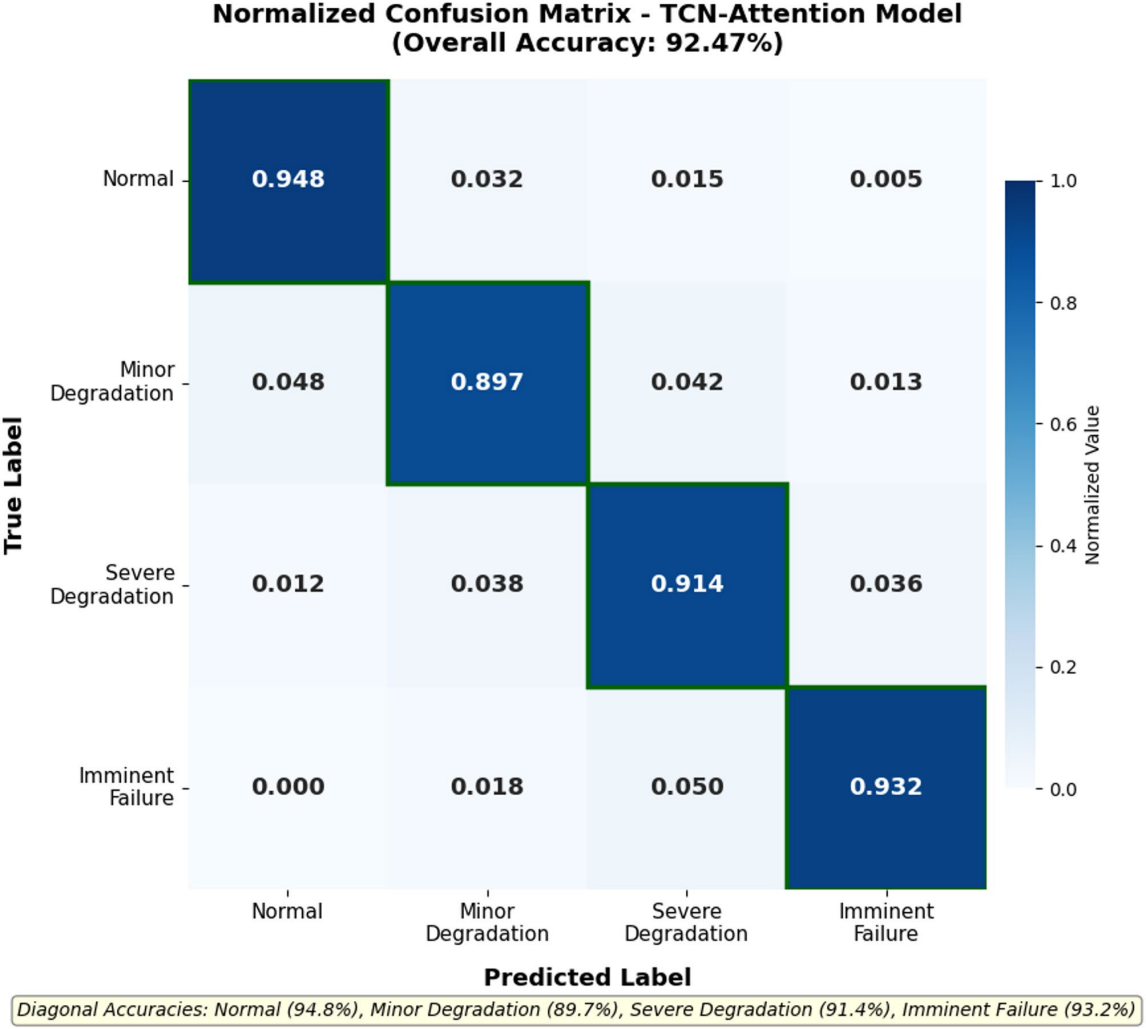
Normalized confusion matrix heatmap for the proposed TCN-Attention model. Diagonal values represent per-class classification accuracy: Normal (94.8%), Minor Degradation (89.7%), Severe Degradation (91.4%), and Imminent Failure (93.2%). The most frequent off-diagonal entry corresponds to Minor Degradation samples misclassified as Normal (4.8%).

The confusion matrix provides granular insight into classification behavior. Figure 6 displays the normalized confusion matrix for our TCN-Attention model on test data. Diagonal dominance confirms strong overall performance, while off-diagonal elements reveal specific misclassification patterns.

As Fig. 6 demonstrates, the most frequent error involved confusing minor degradation with normal operation (4.8% of minor degradation samples), while misclassifying normal samples as degraded occurred at a lower rate of 2.1%. Severe degradation showed occasional confusion with both adjacent categories, though rarely with normal operation. Notably, no imminent failure sample was ever misclassified as normal. The practical implications of these misclassification patterns are discussed further in Section V.

The weighted kappa coefficient provides an additional perspective accounting for ordinal class relationships:

28 $${\kappa}_{w}=1-\frac{\sum_{i=1}^{K}\sum_{j=1}^{K}{w}_{ij}{O}_{ij}}{\sum_{i=1}^{K}\sum_{j=1}^{K}{w}_{ij}{E}_{ij}}$$

where $K$ is the number of health state categories, ${w}_{ij}={\left|i-j\right|}^{2}$ represents quadratic distance-based weights that penalize disagreements between more distant categories, ${O}_{ij}$ denotes observed frequencies in the confusion matrix, and ${E}_{ij}$ indicates expected frequencies under the assumption of independence between predicted and actual labels.

Our model achieved ${\kappa}_{w}=0.914$, indicating near-perfect agreement when accounting for ordinal severity relationships^56^. To assess statistical significance, we conducted paired t-tests comparing our method against each baseline across the five experimental runs. The improvements over Transformer ($p=0.003$), Informer ($p=0.007$), Autoformer ($p=0.012$), and CATA-TCN ($p=0.024$) were all statistically significant at the 0.05 level. We further examined performance consistency across different equipment units by computing per-unit accuracy: the standard deviation across the 12 equipment units was 2.34%, indicating reasonable generalization across diverse operational histories. Cross-validation using leave-one-equipment-out evaluation yielded mean accuracy of 91.23% (± 1.87%), confirming that the model does not overfit to specific equipment signatures.

### Remaining useful life estimation performance evaluation

Accurate RUL estimation provides maintenance planners with quantitative guidance for scheduling interventions before equipment failure occurs. We evaluated prediction performance using three complementary metrics: RMSE, MAE, and the coefficient of determination (${R}^{2}$). While RMSE penalizes larger errors more heavily due to squaring, MAE offers direct interpretability in original units (operating hours). The ${R}^{2}$ coefficient indicates what proportion of variance in actual RUL values the model successfully explains.

Table 6 compares RUL estimation errors across all evaluated methods. Lower RMSE and MAE values indicate superior prediction accuracy, while higher ${R}^{2}$ values signify better fit quality. We also report the asymmetric score defined earlier, which penalizes late predictions more severely than early ones given the greater practical consequence of unexpected failures.

**Table 6:** RUL estimation error comparison.

Method	RMSE (hours)	MAE (hours)	*R*²	Asymmetric score
SVR	187.34 ± 12.56	142.67 ± 9.84	0.712	3847.2
Random Forest	165.28 ± 10.23	128.45 ± 8.67	0.758	3256.8
LSTM	134.56 ± 8.45	98.23 ± 6.78	0.834	2187.4
Standard TCN	121.34 ± 7.12	87.56 ± 5.92	0.862	1876.3
CNN-LSTM	128.67 ± 7.89	92.34 ± 6.23	0.849	2034.5
Transformer	115.23 ± 6.78	82.45 ± 5.34	0.878	1654.2
Informer^58^	112.45 ± 6.34	80.12 ± 5.12	0.884	1598.7
Autoformer^60^	108.67 ± 5.98	77.89 ± 4.89	0.891	1487.3
CATA-TCN^55^	105.23 ± 5.67	75.34 ± 4.67	0.897	1412.8
TCN-Attention (ours)	98.45 ± 5.23	71.28 ± 4.56	0.912	1287.6

The results in Table 6 demonstrate substantial performance gains achieved by our proposed approach. The TCN-Attention model attained RMSE of 98.45 h and MAE of 71.28 h—reductions of approximately 14.6% and 13.5% compared to the Transformer baseline. Perhaps more importantly, the ${R}^{2}$ value of 0.912 indicates that our model captures over 91% of variance in actual RUL values, a notable improvement over competing methods^57^.

**Fig. 7:**
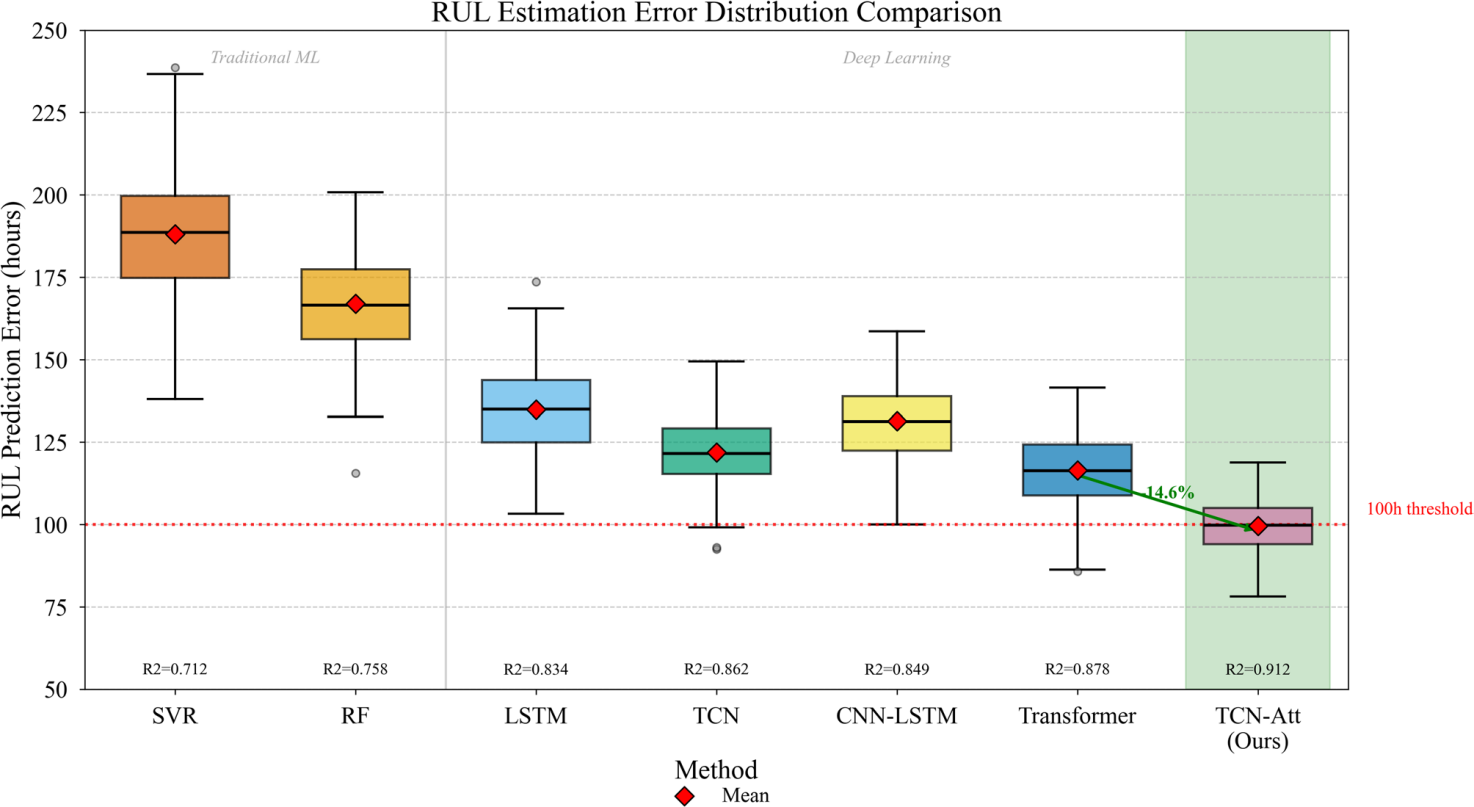
RUL estimation error distribution comparison across different methods.

Figure 7 visualizes prediction error distributions through box plots, enabling comparison of error spread and outlier characteristics across methods. Our approach exhibits not only lower median error but also substantially reduced variance, suggesting more consistent prediction quality across diverse operating conditions.

Prediction accuracy varies considerably depending on where equipment stands in its degradation trajectory. Early-stage predictions—when substantial RUL remains—inherently carry greater uncertainty since numerous factors could influence future degradation rates. We partitioned test samples into three degradation phases based on remaining life percentage: early stage (> 66% RUL remaining), middle stage (33–66%), and late stage (< 33%). The phase-specific RMSE values for our model were 142.3, 87.6, and 54.2 h respectively.

**Fig. 8:**
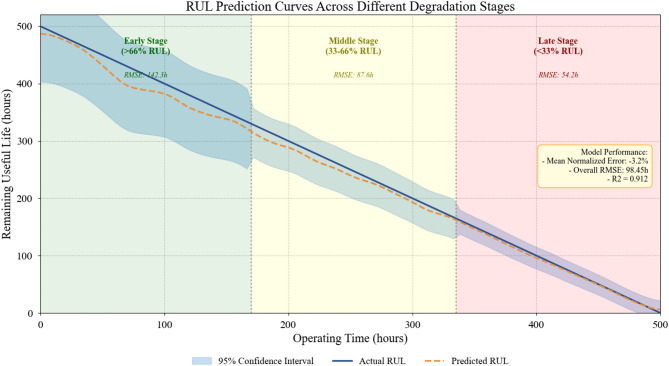
RUL prediction curves across different degradation stages for representative equipment units.

The observed trend—decreasing RMSE as equipment progresses through degradation—is consistent with prior findings reported in^58^. A detailed interpretation of this phase-dependent behavior is provided in Section V. Figure 8 illustrates predicted versus actual RUL trajectories for representative equipment units, with shaded regions indicating prediction uncertainty bounds computed as:

29 $$C{I}_{95\%}\hat ={RUL}\pm1.96\times{\sigma}_{pred}$$

where ${\sigma}_{pred}$ represents the standard deviation of predictions from ensemble runs.

As Fig. 8 demonstrates, predicted trajectories closely track actual values throughout the degradation process, with uncertainty bands appropriately narrowing as failure approaches. The model successfully captures both gradual wear patterns and more rapid deterioration phases that occasionally occur during heavy operational periods.

Ablation experiments systematically quantified each architectural component’s contribution to overall performance. We constructed four model variants: (a) TCN backbone only, (b) TCN with temporal attention, (c) TCN with channel attention, and (d) complete TCN-Attention with both attention types. The incremental RMSE reductions were 121.34→108.56→112.78→98.45 h, confirming that both attention mechanisms provide meaningful improvements. Interestingly, temporal attention contributed slightly more than channel attention in isolation, though their combination yielded synergistic benefits exceeding individual contributions. The multi-task learning framework further reduced RMSE by approximately 8% compared to single-task RUL estimation, validating the value of shared representations between fault prediction and life estimation objectives.

Robustness testing examined model stability under degraded data conditions. We artificially introduced sensor dropout (randomly masking 10–30% of input channels) and measurement noise (Gaussian perturbations with signal-to-noise ratio (SNR) from 20 to 10 dB). Model performance degrades gracefully under these perturbations, with RMSE increasing to 112.3 h at 20% dropout and 118.7 h at 15 dB SNR—still outperforming baseline methods under clean conditions. This resilience stems partly from the attention mechanism’s ability to adaptively downweight corrupted or noisy channels^59^. We computed the degradation ratio as:

30 $${\eta}_{robust}=\frac{RMS{E}_{perturbed}-RMS{E}_{clean}}{RMS{E}_{clean}}\times100\%$$

Our model achieved ${\eta}_{robust}=14.1\%$ at 20% dropout, substantially lower than LSTM (23.7%) and standard TCN (19.2%), confirming enhanced robustness to practical data quality issues.

The percentage of predictions falling within acceptable error bounds provides practical performance insight. Defining acceptable predictions as those within $\pm15\%$ of actual RUL, our model achieved 84.6% acceptable rate compared to 76.2% for Transformer and 71.8% for standard TCN. This metric directly relates to maintenance scheduling reliability—higher acceptable rates translate to more dependable planning horizons. The normalized prediction error distribution follows:

31 $${e}_{norm}=\frac{RU{L}_{pred}-RU{L}_{true}}{RU{L}_{true}}\times100\%$$

Analysis revealed a slight systematic bias toward conservative (early) predictions, with mean normalized error of − 3.2%. The practical significance of this tendency is examined in Section V.

## Discussion

Several patterns in the fault classification results deserve closer examination here. The confusion matrix revealed that the most common misclassification involved labeling minor degradation as normal operation—a direction that, in practice, could delay necessary maintenance interventions. This type of error is more consequential than the reverse (labeling normal as degraded), because the latter merely triggers an unnecessary inspection rather than a missed fault. We find it reassuring, however, that imminent failure samples were never confused with normal operation, which means the model reliably flags the most safety-critical conditions. Similar misclassification asymmetries have been documented in bearing fault diagnosis studies, where adjacent health states consistently prove harder to distinguish than states at opposite ends of the severity spectrum^55^.

Regarding phase-dependent RUL accuracy, the observation that early-stage predictions carry higher RMSE aligns well with physical reasoning. In the initial phase of degradation, wear proceeds gradually and signal variations remain subtle—conditions that inherently limit long-range forecasting precision. As a component approaches end-of-life, degradation signatures grow more pronounced and distinctive, enabling tighter prediction intervals^58^. This phase-dependent pattern has also been reported in turbofan engine prognostics on the C-MAPSS benchmark, where RMSE values for early-life predictions typically exceed late-life values by a factor of two or more^35^. Our model’s conservative bias (mean normalized error of − 3.2%) represents another noteworthy result from a deployment standpoint. Slightly underestimating remaining life means that scheduled maintenance will, on average, occur before the actual failure—a property well suited to risk-averse maintenance philosophies in safety-critical mining operations.

To place our results in broader context, we compared the magnitude of performance gains against published benchmarks in related domains. For aircraft engine RUL prediction on the C-MAPSS dataset, Kong et al^8^. reported that their CATA-TCN architecture reduced RMSE by approximately 8–12% over standard TCN baselines. Our dual-branch attention design yielded a comparable 18.9% RMSE reduction relative to the standard TCN on mining equipment data (from 121.34 to 98.45 h), suggesting that the simultaneous temporal-channel attention mechanism extracts richer degradation features than single-branch alternatives. The Informer^11^ and Autoformer^60^architectures, originally developed for long-sequence forecasting, showed competitive but not superior performance on our dataset—likely because the ProbSparse and Auto-Correlation mechanisms were optimized for forecasting horizons much longer than those typical in equipment prognostics. Li et al^16^. demonstrated that fusing spatial-temporal information from multiple sensors with prior knowledge can improve RUL accuracy under varying operational conditions, a finding broadly consistent with our channel attention results. These cross-domain comparisons suggest that the performance advantages we observed are not artifacts of dataset-specific tuning but reflect genuine architectural benefits of dual-branch attention in degradation modeling.

The experimental results presented in the preceding sections demonstrate substantial performance improvements achieved by the proposed TCN-Attention fusion model over existing approaches. Understanding the sources of these gains provides valuable insight for future research directions and practical deployment considerations.

The superior performance of our model stems fundamentally from the complementary strengths of its constituent components. Temporal convolutional networks excel at extracting local patterns and progressively building hierarchical representations through stacked dilated convolutions. Yet TCNs alone treat all temporal positions and sensor channels with uniform importance—an assumption that poorly reflects the reality of equipment degradation processes. Certain time windows contain far more diagnostic information than others; similarly, specific sensors prove more indicative of particular fault modes. The attention mechanism addresses precisely this limitation by learning adaptive weighting schemes that emphasize informative elements while suppressing noise.

Adaptability across varying operational conditions represents a persistent challenge in industrial prognostics. Mining equipment experiences dramatic workload fluctuations, environmental variations, and material heterogeneity that can confound naive prediction models. Our approach demonstrates reasonable robustness to such variability, attributable partly to the multi-scale feature fusion strategy that captures patterns at different temporal granularities. Equipment operating under heavier loads exhibits accelerated degradation that manifests at shorter time scales; the model’s ability to simultaneously monitor rapid fluctuations and gradual trends enables appropriate responses to diverse operating regimes.

From an operational perspective, the practical value of accurate fault prediction and RUL estimation extends well beyond maintenance scheduling alone. Reliable predictions feed into spare parts inventory optimization, trimming both stockout risks and carrying costs simultaneously. Production planners gain the ability to fold equipment availability forecasts into their scheduling, which reduces disruptions across mining operations. Early warning of impending failures also strengthens workplace safety by heading off catastrophic breakdowns that could put personnel at risk.

The interpretability afforded by our model carries practical weight for field deployment. Attention weights, once visualized, let maintenance engineers trace which historical periods shaped a given prediction—whether it was a recent episode of operational overload or a long, gradual wear trend that drove the assessment. In our experiments, vibration sensors at gearbox and final drive locations consistently drew high attention weights during late-stage degradation, a finding that resonates with experienced engineers’ intuition about critical failure points. Temperature channels gained prominence when thermal-stress failures were developing, and hydraulic pressure signals dominated during pump degradation episodes. Channel attention weights of this kind could inform sensor placement optimization in future equipment installations, directing instrumentation budgets toward the most diagnostically productive measurement points. These observations parallel findings by Chen et al^7^., who reported that transfer learning approaches for bearing RUL prediction benefit from identifying which sensor channels carry the highest predictive relevance across domains. Kong et al^8^. likewise noted that channel-level attention in their CATA-TCN framework concentrated on a small subset of sensors during advanced degradation, corroborating the idea that attention-based feature selection mirrors expert reasoning. One limitation we should acknowledge is that attention weight inspection provides correlational rather than causal insights—high attention on a particular sensor does not necessarily mean that sensor measures the root cause of failure. Nevertheless, we believe this level of transparency represents a meaningful step beyond purely opaque prediction pipelines.

Practical deployment considerations include computational requirements, data infrastructure, and maintenance workflow integration. Our model requires approximately 14.8 milliseconds per inference on GPU hardware, enabling real-time monitoring at sub-second intervals if needed. For edge deployment scenarios without GPU access, model quantization and pruning techniques could reduce computational demands at modest accuracy cost. The memory footprint of 47 MB accommodates deployment on industrial computing platforms commonly available at mining sites. Data infrastructure requirements include reliable sensor connectivity, preprocessing pipelines for handling missing values and outliers, and secure data transmission protocols. Integration with existing maintenance management systems would require API development for prediction delivery and alert generation based on configurable RUL thresholds. We recommend establishing site-specific calibration procedures to account for local operational patterns that may differ from training data distributions.

## Conclusion

This paper presented a novel deep learning framework integrating temporal convolutional networks with multi-head attention mechanisms for fault prediction and remaining useful life estimation in large-scale mining equipment. The research addressed critical gaps in existing prognostic methodologies by developing an architecture capable of simultaneously capturing long-range temporal dependencies and adaptively weighting informative features from multivariate sensor streams.

The principal contributions of this work can be summarized as follows. First, we designed a hierarchical TCN backbone employing dilated causal convolutions that efficiently extract multi-scale temporal patterns without the computational overhead and gradient instability associated with recurrent architectures. Second, a dual-branch attention module was developed to perform adaptive feature selection along both temporal and channel dimensions, enabling the model to focus on diagnostically relevant signal segments while suppressing noise interference. Third, the multi-task learning framework with uncertainty-based loss weighting allows simultaneous optimization of fault classification and RUL regression objectives, promoting shared feature representations that enhance both tasks.

Experimental validation on real-world mining equipment data demonstrated the effectiveness of our approach. The model achieved 92.47% accuracy in fault prediction—a notable improvement over conventional methods and deep learning baselines. For RUL estimation, RMSE of 98.45 h and coefficient of determination of 0.912 confirmed superior prediction capability. Ablation studies verified that each architectural component contributes meaningfully to overall performance, while robustness testing revealed graceful degradation under challenging data quality conditions.

From a theoretical standpoint, this research advances understanding of how attention mechanisms can complement convolutional temporal modeling in industrial prognostic applications. The learned attention patterns exhibit interpretable behavior that aligns with domain expertise, suggesting the model captures genuine physical relationships rather than spurious correlations. Practically, the framework provides mining enterprises with a deployable solution for predictive maintenance that can reduce unplanned downtime, optimize maintenance scheduling, and enhance operational safety.

Several limitations warrant acknowledgment. The current study focused on specific equipment types from a single mining operation; generalization to different machinery and geological contexts requires further validation. The model assumes availability of continuous sensor measurements, yet real-world deployments often contend with intermittent connectivity and data gaps. Additionally, rare fault modes with limited training examples remain challenging to detect reliably.

Future research directions include extending the framework to incorporate physics-informed constraints that could improve extrapolation beyond observed degradation patterns. Transfer learning strategies merit exploration for adapting pretrained models to new equipment types with minimal labeled data. Integration with digital twin platforms would enable real-time simulation and what-if analysis for maintenance decision support. Finally, uncertainty quantification methods could provide confidence intervals around predictions, empowering operators to make risk-informed maintenance choices.

## Supplementary Information

Below is the link to the electronic supplementary material.

Supplementary Material 1

## Data Availability

The datasets supporting the conclusions of this article are included within the supplementary materials. The supplementary files contain the processed sensor features, health state labels, and RUL ground truth values used in all experiments. Raw vibration signals are available from the corresponding author upon reasonable request due to file size constraints and proprietary considerations.

## Abbreviations

RUL: Remaining useful life
TCN: Temporal convolutional network
LSTM: Long short-term memory
CNN: Convolutional neural network
SVM: Support vector machine
RF: Random forest
GB: Gradient boosting
SVR: Support vector regression
RMSE: Root mean square error
MAE: Mean absolute error
BN: Batch normalization
GPU: Graphics processing unit
SNR: Signal-to-noise ratio
