# Supplementary material for: A TCN-Attention fusion model for fault prediction and remaining useful life estimation of large-scale mining equipment
Source: Sci Rep. 2026 Mar 17;16:13746. doi: 10.1038/s41598-026-43145-z (PMC13125494; doi:10.1038/s41598-026-43145-z)
Supplement: Supplementary file 1 — Supplementary Material 1 [file 41598_2026_43145_MOESM1_ESM.docx]

# Supplementary Materials

## A TCN-Attention Fusion Model for Fault Prediction and Remaining Useful Life Estimation of Large-Scale Mining Equipment

## Supplementary Tables

### Table S1. Detailed Hyperparameter Configuration for Model Training

| **Category** | **Parameter** | **Value** | **Search Range** |
| --- | --- | --- | --- |
| **Network Architecture** | Input sequence length | 256 | [128, 256, 512] |
|  | Number of TCN blocks | 6 | [4, 6, 8] |
|  | Initial channel width | 64 | [32, 64, 128] |
|  | Channel expansion factor | 2 | [1.5, 2, 2.5] |
|  | Kernel size | 3 | [3, 5, 7] |
|  | Number of attention heads | 8 | [4, 8, 16] |
|  | Attention key dimension | 64 | [32, 64, 128] |
|  | Dropout rate | 0.2 | [0.1, 0.2, 0.3, 0.4] |
| **Training Configuration** | Batch size | 64 | [32, 64, 128] |
|  | Initial learning rate | 1e-3 | [1e-4, 5e-4, 1e-3, 5e-3] |
|  | Learning rate scheduler | Cosine annealing | - |
|  | Minimum learning rate | 1e-6 | - |
|  | Optimizer | Adam | [Adam, AdamW, SGD] |
|  | Weight decay | 1e-4 | [1e-5, 1e-4, 1e-3] |
|  | Maximum epochs | 200 | - |
|  | Early stopping patience | 15 | [10, 15, 20] |
| **Loss Weights** | Classification weight (λ₁) | 1.0 | [0.5, 1.0, 2.0] |
|  | Regression weight (λ₂) | 0.5 | [0.25, 0.5, 1.0] |
|  | Regularization weight (λ₃) | 1e-4 | [1e-5, 1e-4, 1e-3] |

### Table S2. Detailed Sensor Channel Configuration

| **Sensor Type** | **Channel ID** | **Sampling Rate** | **Unit** | **Haul Truck** | **Excavator** |
| --- | --- | --- | --- | --- | --- |
| Vibration (X-axis) | V1-V3 | 1 kHz | g | ✓ | ✓ |
| Vibration (Y-axis) | V4-V6 | 1 kHz | g | ✓ | ✓ |
| Vibration (Z-axis) | V7-V9 | 1 kHz | g | ✓ | ✓ |
| Temperature (Engine) | T1 | 10 Hz | °C | ✓ | ✓ |
| Temperature (Gearbox) | T2 | 10 Hz | °C | ✓ | ✓ |
| Temperature (Hydraulic) | T3 | 10 Hz | °C | ✓ | ✓ |
| Pressure (Hydraulic) | P1-P2 | 10 Hz | MPa | ✓ | ✓ |
| Pressure (Engine Oil) | P3 | 10 Hz | MPa | ✓ | ✓ |
| Engine Speed | RPM | 10 Hz | rpm | ✓ | ✓ |
| Load Tonnage | LOAD | 10 Hz | ton | ✓ | - |
| Fuel Consumption | FUEL | 10 Hz | L/h | ✓ | ✓ |
| Boom Position | BP1-BP2 | 10 Hz | degree | - | ✓ |
| Bucket Force | BF | 10 Hz | kN | - | ✓ |

### Table S3. Per-Class Fault Prediction Performance

| **Health State** | **Samples** | **Precision (%)** | **Recall (%)** | **F1-Score (%)** | **Support** |
| --- | --- | --- | --- | --- | --- |
| Normal Operation | 3,245 | 94.23±0.82 | 95.12±0.69 | 94.67±0.71 | 3,245 |
| Minor Degradation | 1,876 | 88.45±1.12 | 86.78±1.24 | 87.60±1.09 | 1,876 |
| Severe Degradation | 1,423 | 90.12±0.95 | 88.94±1.08 | 89.52±0.98 | 1,423 |
| Imminent Failure | 674 | 92.14±0.89 | 91.42±0.94 | 91.78±0.86 | 674 |
| **Macro Average** | **7,218** | **91.23±0.78** | **90.56±0.71** | **90.88±0.69** | **7,218** |
| **Weighted Average** | **7,218** | **91.87±0.72** | **92.47±0.65** | **92.15±0.67** | **7,218** |

### Table S4. Equipment-Specific Performance Analysis

| **Equipment Type** | **Unit ID** | **Fault Accuracy (%)** | **RUL RMSE (hours)** | **RUL MAE (hours)** | **R²** |
| --- | --- | --- | --- | --- | --- |
| **Haul Truck** | HT-001 | 93.24±0.78 | 95.67±5.12 | 68.34±4.23 | 0.921 |
|  | HT-002 | 91.87±0.92 | 102.34±5.89 | 74.56±4.67 | 0.903 |
|  | HT-003 | 92.56±0.85 | 98.12±5.34 | 71.23±4.45 | 0.915 |
|  | HT-004 | 93.12±0.81 | 94.56±5.01 | 67.89±4.12 | 0.924 |
|  | HT-005 | 91.45±0.96 | 103.78±6.12 | 75.34±4.89 | 0.898 |
|  | HT-006 | 92.78±0.84 | 97.45±5.28 | 70.67±4.38 | 0.917 |
|  | HT-007 | 93.01±0.79 | 96.23±5.18 | 69.45±4.28 | 0.919 |
| **Hydraulic Excavator** | EX-001 | 92.34±0.87 | 99.87±5.56 | 72.34±4.52 | 0.908 |
|  | EX-002 | 91.67±0.94 | 101.45±5.78 | 73.89±4.64 | 0.901 |
|  | EX-003 | 92.89±0.82 | 97.23±5.23 | 70.12±4.34 | 0.916 |
|  | EX-004 | 91.23±0.98 | 104.56±6.23 | 76.23±4.95 | 0.894 |
|  | EX-005 | 92.45±0.86 | 98.89±5.45 | 71.56±4.48 | 0.912 |
| **Overall Average** | - | **92.47±0.65** | **98.45±5.23** | **71.28±4.56** | **0.912** |

### Table S5. Ablation Study Results

| **Model Variant** | **Fault Acc (%)** | **Fault F1 (%)** | **RUL RMSE (h)** | **RUL MAE (h)** | **R²** | **Params (M)** |
| --- | --- | --- | --- | --- | --- | --- |
| TCN Only | 88.23±0.87 | 86.19±0.94 | 121.34±7.12 | 87.56±5.92 | 0.862 | 2.14 |
| TCN + Temporal Attention | 90.56±0.74 | 88.92±0.81 | 108.56±6.23 | 78.34±5.12 | 0.889 | 2.67 |
| TCN + Channel Attention | 89.78±0.79 | 88.12±0.86 | 112.78±6.56 | 81.23±5.34 | 0.878 | 2.58 |
| TCN + Dual Attention | 91.89±0.69 | 90.24±0.74 | 103.45±5.67 | 74.56±4.78 | 0.901 | 3.12 |
| TCN + Dual Attention + Multi-scale | 92.47±0.65 | 90.88±0.69 | 98.45±5.23 | 71.28±4.56 | 0.912 | 3.45 |
| w/o Multi-task Learning | 91.23±0.78 | 89.56±0.82 | 107.23±6.12 | 77.45±5.02 | 0.886 | 3.45 |
| w/o Uncertainty Weighting | 91.67±0.72 | 90.12±0.76 | 104.89±5.89 | 75.67±4.89 | 0.894 | 3.45 |
| w/o Residual Connections | 89.45±0.91 | 87.78±0.98 | 115.67±6.78 | 83.45±5.56 | 0.871 | 3.42 |

### Table S6. Robustness Analysis Under Sensor Dropout

| **Dropout Rate (%)** | **Fault Accuracy (%)** | **Fault F1 (%)** | **RUL RMSE (hours)** | **R²** | **Degradation Ratio (%)** |
| --- | --- | --- | --- | --- | --- |
| 0 (Baseline) | 92.47±0.65 | 90.88±0.69 | 98.45±5.23 | 0.912 | - |
| 5 | 91.89±0.72 | 90.23±0.78 | 102.34±5.56 | 0.904 | 3.95 |
| 10 | 91.12±0.81 | 89.45±0.87 | 107.56±5.89 | 0.893 | 9.25 |
| 15 | 90.23±0.89 | 88.56±0.95 | 110.23±6.12 | 0.884 | 11.96 |
| 20 | 89.34±0.98 | 87.67±1.04 | 112.34±6.34 | 0.876 | 14.11 |
| 25 | 88.12±1.12 | 86.45±1.18 | 116.78±6.67 | 0.862 | 18.62 |
| 30 | 86.78±1.24 | 85.12±1.31 | 121.45±7.01 | 0.848 | 23.36 |

### Table S7. Robustness Analysis Under Measurement Noise

| **SNR (dB)** | **Fault Accuracy (%)** | **Fault F1 (%)** | **RUL RMSE (hours)** | **R²** | **Degradation Ratio (%)** |
| --- | --- | --- | --- | --- | --- |
| Clean (Baseline) | 92.47±0.65 | 90.88±0.69 | 98.45±5.23 | 0.912 | - |
| 25 | 92.12±0.69 | 90.56±0.74 | 100.23±5.34 | 0.908 | 1.81 |
| 20 | 91.56±0.75 | 89.98±0.81 | 104.56±5.56 | 0.899 | 6.21 |
| 15 | 90.45±0.84 | 88.89±0.91 | 118.67±6.12 | 0.875 | 20.54 |
| 10 | 88.23±0.98 | 86.67±1.05 | 128.45±6.78 | 0.852 | 30.47 |
| 5 | 84.56±1.23 | 82.89±1.31 | 145.67±7.56 | 0.812 | 47.96 |

### Table S8. Performance at Different Degradation Stages

| **Degradation Stage** | **RUL Range (%)** | **Samples** | **RMSE (hours)** | **MAE (hours)** | **R²** | **Within ±15% (%)** |
| --- | --- | --- | --- | --- | --- | --- |
| Early Stage | >66% | 2,456 | 142.34±8.23 | 108.56±6.78 | 0.856 | 72.34 |
| Middle Stage | 33-66% | 2,678 | 87.56±5.12 | 63.45±4.23 | 0.923 | 86.78 |
| Late Stage | <33% | 2,084 | 54.23±3.45 | 38.67±2.89 | 0.958 | 94.56 |
| **Overall** | 0-100% | **7,218** | **98.45±5.23** | **71.28±4.56** | **0.912** | **84.56** |

### Table S9. Comparison of Different Sequence Lengths

| **Sequence Length** | **Fault Acc (%)** | **RUL RMSE (h)** | **R²** | **Training Time (min)** | **Inference Time (ms)** |
| --- | --- | --- | --- | --- | --- |
| 64 | 89.12±0.92 | 118.34±6.89 | 0.871 | 45 | 8.2 |
| 128 | 91.23±0.78 | 106.45±5.89 | 0.894 | 67 | 10.5 |
| 256 | 92.47±0.65 | 98.45±5.23 | 0.912 | 98 | 14.8 |
| 512 | 92.89±0.62 | 96.78±5.01 | 0.918 | 156 | 23.4 |
| 1024 | 93.12±0.59 | 95.12±4.89 | 0.921 | 278 | 42.1 |

### Table S10. Attention Weight Statistics Across Health States

| **Health State** | **Temporal Attention Entropy** | **Channel Attention Entropy** | **Top-3 Temporal Focus (%)** | **Top-3 Channel Focus (%)** |
| --- | --- | --- | --- | --- |
| Normal Operation | 3.84±0.23 | 2.56±0.18 | 28.45±2.34 | 42.34±3.12 |
| Minor Degradation | 3.12±0.28 | 2.23±0.21 | 35.67±2.89 | 48.56±3.45 |
| Severe Degradation | 2.67±0.32 | 1.89±0.24 | 42.34±3.12 | 54.23±3.78 |
| Imminent Failure | 2.12±0.38 | 1.45±0.28 | 51.23±3.56 | 62.45±4.12 |

### Table S11. Computational Resource Requirements

| **Metric** | **Training** | **Inference** |
| --- | --- | --- |
| GPU Memory Usage | 8.4 GB | 2.1 GB |
| CPU Memory Usage | 12.6 GB | 4.2 GB |
| Training Time (200 epochs) | 98 minutes | - |
| Inference Time (per sample) | - | 14.8 ms |
| Model Size | 13.8 MB | 13.8 MB |
| Total Parameters | 3.45 M | 3.45 M |
| Trainable Parameters | 3.45 M | - |
| FLOPs (per inference) | 287.4 M | 287.4 M |

### Table S12. Cross-Validation Results (5-Fold)

| **Fold** | **Fault Accuracy (%)** | **Fault F1 (%)** | **RUL RMSE (hours)** | **RUL MAE (hours)** | **R²** |
| --- | --- | --- | --- | --- | --- |
| Fold 1 | 92.34 | 90.67 | 99.12 | 71.89 | 0.910 |
| Fold 2 | 92.78 | 91.23 | 97.45 | 70.34 | 0.915 |
| Fold 3 | 91.89 | 90.12 | 100.23 | 72.56 | 0.906 |
| Fold 4 | 92.56 | 90.89 | 98.34 | 71.12 | 0.913 |
| Fold 5 | 92.78 | 91.45 | 97.12 | 70.45 | 0.916 |
| **Mean±Std** | **92.47±0.36** | **90.87±0.52** | **98.45±1.23** | **71.27±0.89** | **0.912±0.004** |

### Table S13. Feature Importance Ranking Based on Channel Attention Weights

| **Rank** | **Feature** | **Average Attention Weight** | **Std** | **Category** |
| --- | --- | --- | --- | --- |
| 1 | Vibration (Z-axis, Gearbox) | 0.0823 | 0.0089 | Vibration |
| 2 | Temperature (Gearbox) | 0.0756 | 0.0078 | Temperature |
| 3 | Vibration (X-axis, Bearing) | 0.0712 | 0.0082 | Vibration |
| 4 | Pressure (Hydraulic-1) | 0.0689 | 0.0075 | Pressure |
| 5 | Vibration (Y-axis, Gearbox) | 0.0645 | 0.0081 | Vibration |
| 6 | Temperature (Hydraulic) | 0.0623 | 0.0072 | Temperature |
| 7 | Engine Speed | 0.0598 | 0.0068 | Operational |
| 8 | Vibration (Z-axis, Bearing) | 0.0567 | 0.0079 | Vibration |
| 9 | Pressure (Hydraulic-2) | 0.0534 | 0.0071 | Pressure |
| 10 | Temperature (Engine) | 0.0512 | 0.0065 | Temperature |

## Supplementary Code

### Code S1. TCN-Attention Model Implementation (PyTorch)

import torch
import torch.nn as nn
import torch.nn.functional as F
from torch.nn.utils import weight_norm
import math

class Chomp1d(nn.Module):
 """Remove future time steps to ensure causal convolution."""
 def __init__(self, chomp_size):
 super(Chomp1d, self).__init__()
 self.chomp_size = chomp_size

 def forward(self, x):
 return x[:, :, :-self.chomp_size].contiguous()


class TemporalBlock(nn.Module):
 """Single TCN block with dilated causal convolution and residual connection."""
 def __init__(self, n_inputs, n_outputs, kernel_size, stride, dilation, padding, dropout=0.2):
 super(TemporalBlock, self).__init__()

 self.conv1 = weight_norm(nn.Conv1d(
 n_inputs, n_outputs, kernel_size,
 stride=stride, padding=padding, dilation=dilation
 ))
 self.chomp1 = Chomp1d(padding)
 self.relu1 = nn.ReLU()
 self.dropout1 = nn.Dropout(dropout)

 self.conv2 = weight_norm(nn.Conv1d(
 n_outputs, n_outputs, kernel_size,
 stride=stride, padding=padding, dilation=dilation
 ))
 self.chomp2 = Chomp1d(padding)
 self.relu2 = nn.ReLU()
 self.dropout2 = nn.Dropout(dropout)

 self.net = nn.Sequential(
 self.conv1, self.chomp1, self.relu1, self.dropout1,
 self.conv2, self.chomp2, self.relu2, self.dropout2
 )

 # Residual connection with 1x1 convolution if dimensions differ
 self.downsample = nn.Conv1d(n_inputs, n_outputs, 1) if n_inputs != n_outputs else None
 self.relu = nn.ReLU()
 self.init_weights()

 def init_weights(self):
 self.conv1.weight.data.normal_(0, 0.01)
 self.conv2.weight.data.normal_(0, 0.01)
 if self.downsample is not None:
 self.downsample.weight.data.normal_(0, 0.01)

 def forward(self, x):
 out = self.net(x)
 res = x if self.downsample is None else self.downsample(x)
 return self.relu(out + res)


class TemporalConvNet(nn.Module):
 """Temporal Convolutional Network backbone."""
 def __init__(self, num_inputs, num_channels, kernel_size=3, dropout=0.2):
 super(TemporalConvNet, self).__init__()
 layers = []
 num_levels = len(num_channels)

 for i in range(num_levels):
 dilation_size = 2 ** i
 in_channels = num_inputs if i == 0 else num_channels[i-1]
 out_channels = num_channels[i]
 layers.append(TemporalBlock(
 in_channels, out_channels, kernel_size,
 stride=1, dilation=dilation_size,
 padding=(kernel_size-1) * dilation_size,
 dropout=dropout
 ))

 self.network = nn.Sequential(*layers)

 def forward(self, x):
 return self.network(x)


class MultiHeadAttention(nn.Module):
 """Multi-head self-attention mechanism."""
 def __init__(self, d_model, num_heads, dropout=0.1):
 super(MultiHeadAttention, self).__init__()
 assert d_model % num_heads == 0

 self.d_model = d_model
 self.num_heads = num_heads
 self.d_k = d_model // num_heads

 self.W_q = nn.Linear(d_model, d_model)
 self.W_k = nn.Linear(d_model, d_model)
 self.W_v = nn.Linear(d_model, d_model)
 self.W_o = nn.Linear(d_model, d_model)

 self.dropout = nn.Dropout(dropout)
 self.scale = math.sqrt(self.d_k)

 def forward(self, query, key, value, mask=None):
 batch_size = query.size(0)

 # Linear projections
 Q = self.W_q(query).view(batch_size, -1, self.num_heads, self.d_k).transpose(1, 2)
 K = self.W_k(key).view(batch_size, -1, self.num_heads, self.d_k).transpose(1, 2)
 V = self.W_v(value).view(batch_size, -1, self.num_heads, self.d_k).transpose(1, 2)

 # Scaled dot-product attention
 scores = torch.matmul(Q, K.transpose(-2, -1)) / self.scale

 if mask is not None:
 scores = scores.masked_fill(mask == 0, -1e9)

 attn_weights = F.softmax(scores, dim=-1)
 attn_weights = self.dropout(attn_weights)

 # Apply attention to values
 context = torch.matmul(attn_weights, V)

 # Concatenate heads and apply output projection
 context = context.transpose(1, 2).contiguous().view(batch_size, -1, self.d_model)
 output = self.W_o(context)

 return output, attn_weights


class ChannelAttention(nn.Module):
 """Channel-wise attention module."""
 def __init__(self, num_channels, reduction_ratio=8):
 super(ChannelAttention, self).__init__()

 self.avg_pool = nn.AdaptiveAvgPool1d(1)
 self.max_pool = nn.AdaptiveMaxPool1d(1)

 self.fc = nn.Sequential(
 nn.Linear(num_channels, num_channels // reduction_ratio, bias=False),
 nn.ReLU(inplace=True),
 nn.Linear(num_channels // reduction_ratio, num_channels, bias=False)
 )
 self.sigmoid = nn.Sigmoid()

 def forward(self, x):
 # x: (batch, channels, seq_len)
 avg_out = self.fc(self.avg_pool(x).squeeze(-1))
 max_out = self.fc(self.max_pool(x).squeeze(-1))
 channel_weights = self.sigmoid(avg_out + max_out).unsqueeze(-1)
 return x * channel_weights, channel_weights.squeeze(-1)


class TemporalAttention(nn.Module):
 """Temporal attention module with temperature scaling."""
 def __init__(self, d_model, temperature=0.5):
 super(TemporalAttention, self).__init__()

 self.temperature = temperature
 self.query = nn.Linear(d_model, d_model)
 self.key = nn.Linear(d_model, d_model)
 self.v = nn.Linear(d_model, 1)

 def forward(self, x):
 # x: (batch, seq_len, d_model)
 q = torch.tanh(self.query(x))
 k = torch.tanh(self.key(x))

 # Compute attention scores
 scores = self.v(q * k).squeeze(-1) # (batch, seq_len)
 attn_weights = F.softmax(scores / self.temperature, dim=-1)

 # Weighted sum
 context = torch.bmm(attn_weights.unsqueeze(1), x).squeeze(1)

 return context, attn_weights


class DualAttentionModule(nn.Module):
 """Combined temporal and channel attention."""
 def __init__(self, d_model, num_heads=8, temperature=0.5, dropout=0.1):
 super(DualAttentionModule, self).__init__()

 self.layer_norm1 = nn.LayerNorm(d_model)
 self.layer_norm2 = nn.LayerNorm(d_model)

 self.multi_head_attn = MultiHeadAttention(d_model, num_heads, dropout)
 self.channel_attn = ChannelAttention(d_model)
 self.temporal_attn = TemporalAttention(d_model, temperature)

 self.dropout = nn.Dropout(dropout)

 def forward(self, x):
 # x: (batch, channels, seq_len)

 # Channel attention
 x_channel, channel_weights = self.channel_attn(x)

 # Transpose for temporal processing: (batch, seq_len, channels)
 x_temporal = x_channel.transpose(1, 2)
 x_temporal = self.layer_norm1(x_temporal)

 # Multi-head self-attention
 attn_out, attn_weights = self.multi_head_attn(x_temporal, x_temporal, x_temporal)
 x_temporal = x_temporal + self.dropout(attn_out)
 x_temporal = self.layer_norm2(x_temporal)

 # Temporal attention for aggregation
 context, temporal_weights = self.temporal_attn(x_temporal)

 return context, temporal_weights, channel_weights


class TCNAttentionModel(nn.Module):
 """Complete TCN-Attention fusion model for fault prediction and RUL estimation."""
 def __init__(
 self,
 input_channels,
 num_classes=4,
 tcn_channels=[64, 64, 128, 128, 256, 256],
 kernel_size=3,
 num_heads=8,
 temperature=0.5,
 dropout=0.2
 ):
 super(TCNAttentionModel, self).__init__()

 self.input_channels = input_channels
 self.num_classes = num_classes

 # TCN backbone
 self.tcn = TemporalConvNet(
 num_inputs=input_channels,
 num_channels=tcn_channels,
 kernel_size=kernel_size,
 dropout=dropout
 )

 # Dual attention module
 self.attention = DualAttentionModule(
 d_model=tcn_channels[-1],
 num_heads=num_heads,
 temperature=temperature,
 dropout=dropout
 )

 # Task-specific output heads
 hidden_dim = tcn_channels[-1]

 # Fault prediction branch
 self.fault_head = nn.Sequential(
 nn.Linear(hidden_dim, hidden_dim // 2),
 nn.ReLU(),
 nn.Dropout(dropout),
 nn.Linear(hidden_dim // 2, num_classes)
 )

 # RUL estimation branch
 self.rul_head = nn.Sequential(
 nn.Linear(hidden_dim, hidden_dim // 2),
 nn.ReLU(),
 nn.Dropout(dropout),
 nn.Linear(hidden_dim // 2, 1)
 )

 # Learnable uncertainty parameters for multi-task learning
 self.log_sigma_fault = nn.Parameter(torch.zeros(1))
 self.log_sigma_rul = nn.Parameter(torch.zeros(1))

 def forward(self, x):
 """
 Forward pass.

 Args:
 x: Input tensor of shape (batch, seq_len, input_channels)

 Returns:
 fault_logits: Classification logits (batch, num_classes)
 rul_pred: RUL prediction (batch, 1)
 attention_weights: Dictionary containing attention weights
 """
 # Transpose for TCN: (batch, channels, seq_len)
 x = x.transpose(1, 2)

 # TCN feature extraction
 tcn_features = self.tcn(x)

 # Dual attention
 context, temporal_weights, channel_weights = self.attention(tcn_features)

 # Task-specific predictions
 fault_logits = self.fault_head(context)
 rul_pred = self.rul_head(context)

 attention_weights = {
 'temporal': temporal_weights,
 'channel': channel_weights
 }

 return fault_logits, rul_pred, attention_weights

 def compute_loss(self, fault_logits, rul_pred, fault_labels, rul_labels):
 """
 Compute multi-task loss with uncertainty weighting.

 Args:
 fault_logits: Predicted fault logits
 rul_pred: Predicted RUL values
 fault_labels: Ground truth fault labels
 rul_labels: Ground truth RUL values

 Returns:
 total_loss: Combined loss value
 loss_dict: Dictionary containing individual loss components
 """
 # Classification loss
 ce_loss = F.cross_entropy(fault_logits, fault_labels)

 # Regression loss
 mse_loss = F.mse_loss(rul_pred.squeeze(), rul_labels)

 # Uncertainty-weighted combination
 precision_fault = torch.exp(-self.log_sigma_fault)
 precision_rul = torch.exp(-self.log_sigma_rul)

 total_loss = (
 precision_fault * ce_loss + self.log_sigma_fault +
 precision_rul * mse_loss + self.log_sigma_rul
 )

 loss_dict = {
 'total': total_loss.item(),
 'fault_ce': ce_loss.item(),
 'rul_mse': mse_loss.item(),
 'sigma_fault': torch.exp(self.log_sigma_fault).item(),
 'sigma_rul': torch.exp(self.log_sigma_rul).item()
 }

 return total_loss, loss_dict


# Example usage and model instantiation
if __name__ == "__main__":
 # Model configuration
 config = {
 'input_channels': 18, # Number of sensor channels
 'num_classes': 4, # Health states
 'tcn_channels': [64, 64, 128, 128, 256, 256],
 'kernel_size': 3,
 'num_heads': 8,
 'temperature': 0.5,
 'dropout': 0.2
 }

 # Initialize model
 model = TCNAttentionModel(**config)

 # Print model summary
 total_params = sum(p.numel() for p in model.parameters())
 trainable_params = sum(p.numel() for p in model.parameters() if p.requires_grad)
 print(f"Total parameters: {total_params:,}")
 print(f"Trainable parameters: {trainable_params:,}")

 # Test forward pass
 batch_size = 32
 seq_length = 256
 x = torch.randn(batch_size, seq_length, config['input_channels'])

 fault_logits, rul_pred, attn_weights = model(x)

 print(f"\nInput shape: {x.shape}")
 print(f"Fault logits shape: {fault_logits.shape}")
 print(f"RUL prediction shape: {rul_pred.shape}")
 print(f"Temporal attention shape: {attn_weights['temporal'].shape}")
 print(f"Channel attention shape: {attn_weights['channel'].shape}")

### Code S2. Training Pipeline

import torch
import torch.nn as nn
import torch.optim as optim
from torch.utils.data import DataLoader, Dataset
import numpy as np
from sklearn.metrics import accuracy_score, precision_recall_fscore_support
from sklearn.metrics import mean_squared_error, mean_absolute_error, r2_score
import time
from tqdm import tqdm


class MiningEquipmentDataset(Dataset):
 """Custom dataset for mining equipment sensor data."""

 def __init__(self, sequences, fault_labels, rul_labels, transform=None):
 """
 Args:
 sequences: Sensor data sequences (N, T, D)
 fault_labels: Fault category labels (N,)
 rul_labels: RUL values in hours (N,)
 transform: Optional data augmentation
 """
 self.sequences = torch.FloatTensor(sequences)
 self.fault_labels = torch.LongTensor(fault_labels)
 self.rul_labels = torch.FloatTensor(rul_labels)
 self.transform = transform

 def __len__(self):
 return len(self.sequences)

 def __getitem__(self, idx):
 x = self.sequences[idx]

 if self.transform is not None:
 x = self.transform(x)

 return x, self.fault_labels[idx], self.rul_labels[idx]


class CosineAnnealingWarmup:
 """Learning rate scheduler with warmup and cosine annealing."""

 def __init__(self, optimizer, warmup_epochs, max_epochs, min_lr=1e-6):
 self.optimizer = optimizer
 self.warmup_epochs = warmup_epochs
 self.max_epochs = max_epochs
 self.min_lr = min_lr
 self.base_lr = optimizer.param_groups[0]['lr']

 def step(self, epoch):
 if epoch < self.warmup_epochs:
 lr = self.base_lr * (epoch + 1) / self.warmup_epochs
 else:
 progress = (epoch - self.warmup_epochs) / (self.max_epochs - self.warmup_epochs)
 lr = self.min_lr + 0.5 * (self.base_lr - self.min_lr) * (1 + np.cos(np.pi * progress))

 for param_group in self.optimizer.param_groups:
 param_group['lr'] = lr

 return lr


class EarlyStopping:
 """Early stopping handler."""

 def __init__(self, patience=15, min_delta=1e-4, mode='min'):
 self.patience = patience
 self.min_delta = min_delta
 self.mode = mode
 self.counter = 0
 self.best_score = None
 self.early_stop = False

 def __call__(self, score):
 if self.best_score is None:
 self.best_score = score
 elif self._is_improvement(score):
 self.best_score = score
 self.counter = 0
 else:
 self.counter += 1
 if self.counter >= self.patience:
 self.early_stop = True

 return self.early_stop

 def _is_improvement(self, score):
 if self.mode == 'min':
 return score < self.best_score - self.min_delta
 return score > self.best_score + self.min_delta


def train_epoch(model, train_loader, optimizer, device):
 """Train for one epoch."""
 model.train()
 total_loss = 0
 all_fault_preds = []
 all_fault_labels = []
 all_rul_preds = []
 all_rul_labels = []

 for batch_x, batch_fault, batch_rul in tqdm(train_loader, desc="Training"):
 batch_x = batch_x.to(device)
 batch_fault = batch_fault.to(device)
 batch_rul = batch_rul.to(device)

 optimizer.zero_grad()

 fault_logits, rul_pred, _ = model(batch_x)
 loss, _ = model.compute_loss(fault_logits, rul_pred, batch_fault, batch_rul)

 loss.backward()
 torch.nn.utils.clip_grad_norm_(model.parameters(), max_norm=1.0)
 optimizer.step()

 total_loss += loss.item()

 # Collect predictions
 all_fault_preds.extend(fault_logits.argmax(dim=1).cpu().numpy())
 all_fault_labels.extend(batch_fault.cpu().numpy())
 all_rul_preds.extend(rul_pred.squeeze().detach().cpu().numpy())
 all_rul_labels.extend(batch_rul.cpu().numpy())

 # Compute metrics
 fault_acc = accuracy_score(all_fault_labels, all_fault_preds)
 rul_rmse = np.sqrt(mean_squared_error(all_rul_labels, all_rul_preds))

 return total_loss / len(train_loader), fault_acc, rul_rmse


def evaluate(model, val_loader, device):
 """Evaluate model on validation set."""
 model.eval()
 total_loss = 0
 all_fault_preds = []
 all_fault_labels = []
 all_rul_preds = []
 all_rul_labels = []

 with torch.no_grad():
 for batch_x, batch_fault, batch_rul in tqdm(val_loader, desc="Evaluating"):
 batch_x = batch_x.to(device)
 batch_fault = batch_fault.to(device)
 batch_rul = batch_rul.to(device)

 fault_logits, rul_pred, _ = model(batch_x)
 loss, _ = model.compute_loss(fault_logits, rul_pred, batch_fault, batch_rul)

 total_loss += loss.item()

 all_fault_preds.extend(fault_logits.argmax(dim=1).cpu().numpy())
 all_fault_labels.extend(batch_fault.cpu().numpy())
 all_rul_preds.extend(rul_pred.squeeze().cpu().numpy())
 all_rul_labels.extend(batch_rul.cpu().numpy())

 # Compute comprehensive metrics
 fault_acc = accuracy_score(all_fault_labels, all_fault_preds)
 precision, recall, f1, _ = precision_recall_fscore_support(
 all_fault_labels, all_fault_preds, average='macro'
 )

 rul_rmse = np.sqrt(mean_squared_error(all_rul_labels, all_rul_preds))
 rul_mae = mean_absolute_error(all_rul_labels, all_rul_preds)
 rul_r2 = r2_score(all_rul_labels, all_rul_preds)

 metrics = {
 'loss': total_loss / len(val_loader),
 'fault_accuracy': fault_acc,
 'fault_precision': precision,
 'fault_recall': recall,
 'fault_f1': f1,
 'rul_rmse': rul_rmse,
 'rul_mae': rul_mae,
 'rul_r2': rul_r2
 }

 return metrics


def train_model(model, train_loader, val_loader, config, device):
 """Complete training pipeline."""

 optimizer = optim.Adam(
 model.parameters(),
 lr=config['learning_rate'],
 weight_decay=config['weight_decay']
 )

 scheduler = CosineAnnealingWarmup(
 optimizer,
 warmup_epochs=config['warmup_epochs'],
 max_epochs=config['max_epochs'],
 min_lr=config['min_lr']
 )

 early_stopping = EarlyStopping(patience=config['patience'], mode='min')

 best_val_loss = float('inf')
 best_model_state = None
 history = {'train': [], 'val': []}

 for epoch in range(config['max_epochs']):
 start_time = time.time()

 # Update learning rate
 current_lr = scheduler.step(epoch)

 # Training
 train_loss, train_fault_acc, train_rul_rmse = train_epoch(
 model, train_loader, optimizer, device
 )

 # Validation
 val_metrics = evaluate(model, val_loader, device)

 epoch_time = time.time() - start_time

 # Logging
 print(f"\nEpoch {epoch+1}/{config['max_epochs']} ({epoch_time:.1f}s)")
 print(f" LR: {current_lr:.6f}")
 print(f" Train - Loss: {train_loss:.4f}, Fault Acc: {train_fault_acc:.4f}, RUL RMSE: {train_rul_rmse:.2f}")
 print(f" Val - Loss: {val_metrics['loss']:.4f}, Fault Acc: {val_metrics['fault_accuracy']:.4f}, "
 f"F1: {val_metrics['fault_f1']:.4f}, RUL RMSE: {val_metrics['rul_rmse']:.2f}, R²: {val_metrics['rul_r2']:.4f}")

 # Save history
 history['train'].append({
 'loss': train_loss,
 'fault_acc': train_fault_acc,
 'rul_rmse': train_rul_rmse
 })
 history['val'].append(val_metrics)

 # Save best model
 if val_metrics['loss'] < best_val_loss:
 best_val_loss = val_metrics['loss']
 best_model_state = model.state_dict().copy()
 print(" ** New best model saved **")

 # Early stopping check
 if early_stopping(val_metrics['loss']):
 print(f"\nEarly stopping triggered at epoch {epoch+1}")
 break

 # Load best model
 model.load_state_dict(best_model_state)

 return model, history


# Main execution
if __name__ == "__main__":
 # Configuration
 config = {
 'batch_size': 64,
 'learning_rate': 1e-3,
 'weight_decay': 1e-4,
 'max_epochs': 200,
 'warmup_epochs': 10,
 'min_lr': 1e-6,
 'patience': 15
 }

 device = torch.device('cuda' if torch.cuda.is_available() else 'cpu')
 print(f"Using device: {device}")

 # Example: Create synthetic data for demonstration
 # In practice, replace with actual mining equipment data
 np.random.seed(42)
 n_samples = 1000
 seq_length = 256
 n_channels = 18
 n_classes = 4

 X_train = np.random.randn(n_samples, seq_length, n_channels).astype(np.float32)
 y_fault_train = np.random.randint(0, n_classes, n_samples)
 y_rul_train = np.random.uniform(0, 500, n_samples).astype(np.float32)

 X_val = np.random.randn(n_samples // 5, seq_length, n_channels).astype(np.float32)
 y_fault_val = np.random.randint(0, n_classes, n_samples // 5)
 y_rul_val = np.random.uniform(0, 500, n_samples // 5).astype(np.float32)

 # Create datasets and loaders
 train_dataset = MiningEquipmentDataset(X_train, y_fault_train, y_rul_train)
 val_dataset = MiningEquipmentDataset(X_val, y_fault_val, y_rul_val)

 train_loader = DataLoader(train_dataset, batch_size=config['batch_size'], shuffle=True, num_workers=4)
 val_loader = DataLoader(val_dataset, batch_size=config['batch_size'], shuffle=False, num_workers=4)

 # Initialize model
 model_config = {
 'input_channels': n_channels,
 'num_classes': n_classes,
 'tcn_channels': [64, 64, 128, 128, 256, 256],
 'kernel_size': 3,
 'num_heads': 8,
 'temperature': 0.5,
 'dropout': 0.2
 }

 model = TCNAttentionModel(**model_config).to(device)

 # Train model
 trained_model, history = train_model(model, train_loader, val_loader, config, device)

 # Save model
 torch.save({
 'model_state_dict': trained_model.state_dict(),
 'model_config': model_config,
 'training_config': config,
 'history': history
 }, 'tcn_attention_model.pth')

 print("\nTraining completed and model saved!")

### Code S3. Evaluation and Visualization

import torch
import numpy as np
import matplotlib.pyplot as plt
import seaborn as sns
from sklearn.metrics import confusion_matrix, classification_report
from sklearn.metrics import mean_squared_error, mean_absolute_error, r2_score
import pandas as pd


def plot_confusion_matrix(y_true, y_pred, class_names, save_path=None):
 """Plot normalized confusion matrix."""
 cm = confusion_matrix(y_true, y_pred, normalize='true')

 plt.figure(figsize=(10, 8))
 sns.heatmap(cm, annot=True, fmt='.3f', cmap='Blues',
 xticklabels=class_names, yticklabels=class_names)
 plt.xlabel('Predicted Label', fontsize=12)
 plt.ylabel('True Label', fontsize=12)
 plt.title('Normalized Confusion Matrix', fontsize=14)
 plt.tight_layout()

 if save_path:
 plt.savefig(save_path, dpi=300, bbox_inches='tight')
 plt.show()


def plot_rul_predictions(y_true, y_pred, save_path=None):
 """Plot RUL prediction scatter plot."""
 plt.figure(figsize=(10, 8))

 plt.scatter(y_true, y_pred, alpha=0.5, s=20)

 # Perfect prediction line
 min_val = min(min(y_true), min(y_pred))
 max_val = max(max(y_true), max(y_pred))
 plt.plot([min_val, max_val], [min_val, max_val], 'r--', lw=2, label='Perfect Prediction')

 # ±15% bounds
 plt.fill_between([min_val, max_val],
 [min_val * 0.85, max_val * 0.85],
 [min_val * 1.15, max_val * 1.15],
 alpha=0.2, color='green', label='±15% Bounds')

 plt.xlabel('Actual RUL (hours)', fontsize=12)
 plt.ylabel('Predicted RUL (hours)', fontsize=12)
 plt.title('RUL Prediction vs Actual', fontsize=14)
 plt.legend()
 plt.grid(True, alpha=0.3)
 plt.tight_layout()

 if save_path:
 plt.savefig(save_path, dpi=300, bbox_inches='tight')
 plt.show()


def plot_attention_weights(temporal_weights, channel_weights, sensor_names=None, save_path=None):
 """Visualize attention weight distributions."""
 fig, axes = plt.subplots(1, 2, figsize=(14, 5))

 # Temporal attention
 ax1 = axes[0]
 ax1.bar(range(len(temporal_weights)), temporal_weights, color='steelblue', alpha=0.7)
 ax1.set_xlabel('Time Step', fontsize=11)
 ax1.set_ylabel('Attention Weight', fontsize=11)
 ax1.set_title('Temporal Attention Distribution', fontsize=12)
 ax1.grid(True, alpha=0.3)

 # Channel attention
 ax2 = axes[1]
 if sensor_names is None:
 sensor_names = [f'Ch-{i}' for i in range(len(channel_weights))]

 y_pos = np.arange(len(channel_weights))
 ax2.barh(y_pos, channel_weights, color='darkorange', alpha=0.7)
 ax2.set_yticks(y_pos)
 ax2.set_yticklabels(sensor_names, fontsize=9)
 ax2.set_xlabel('Attention Weight', fontsize=11)
 ax2.set_title('Channel Attention Distribution', fontsize=12)
 ax2.grid(True, alpha=0.3)

 plt.tight_layout()

 if save_path:
 plt.savefig(save_path, dpi=300, bbox_inches='tight')
 plt.show()


def plot_training_history(history, save_path=None):
 """Plot training and validation curves."""
 fig, axes = plt.subplots(2, 2, figsize=(14, 10))

 epochs = range(1, len(history['train']) + 1)

 # Loss curves
 ax1 = axes[0, 0]
 ax1.plot(epochs, [h['loss'] for h in history['train']], 'b-', label='Training')
 ax1.plot(epochs, [h['loss'] for h in history['val']], 'r-', label='Validation')
 ax1.set_xlabel('Epoch')
 ax1.set_ylabel('Loss')
 ax1.set_title('Training and Validation Loss')
 ax1.legend()
 ax1.grid(True, alpha=0.3)

 # Fault accuracy curves
 ax2 = axes[0, 1]
 ax2.plot(epochs, [h['fault_acc'] for h in history['train']], 'b-', label='Training')
 ax2.plot(epochs, [h['fault_accuracy'] for h in history['val']], 'r-', label='Validation')
 ax2.set_xlabel('Epoch')
 ax2.set_ylabel('Accuracy')
 ax2.set_title('Fault Prediction Accuracy')
 ax2.legend()
 ax2.grid(True, alpha=0.3)

 # RUL RMSE curves
 ax3 = axes[1, 0]
 ax3.plot(epochs, [h['rul_rmse'] for h in history['train']], 'b-', label='Training')
 ax3.plot(epochs, [h['rul_rmse'] for h in history['val']], 'r-', label='Validation')
 ax3.set_xlabel('Epoch')
 ax3.set_ylabel('RMSE (hours)')
 ax3.set_title('RUL Estimation RMSE')
 ax3.legend()
 ax3.grid(True, alpha=0.3)

 # R² score curves
 ax4 = axes[1, 1]
 ax4.plot(epochs, [h['rul_r2'] for h in history['val']], 'g-', label='Validation R²')
 ax4.set_xlabel('Epoch')
 ax4.set_ylabel('R² Score')
 ax4.set_title('RUL Estimation R² Score')
 ax4.legend()
 ax4.grid(True, alpha=0.3)

 plt.tight_layout()

 if save_path:
 plt.savefig(save_path, dpi=300, bbox_inches='tight')
 plt.show()


def compute_comprehensive_metrics(y_fault_true, y_fault_pred, y_rul_true, y_rul_pred):
 """Compute all evaluation metrics."""

 # Fault prediction metrics
 fault_report = classification_report(y_fault_true, y_fault_pred, output_dict=True)

 # RUL estimation metrics
 rul_rmse = np.sqrt(mean_squared_error(y_rul_true, y_rul_pred))
 rul_mae = mean_absolute_error(y_rul_true, y_rul_pred)
 rul_r2 = r2_score(y_rul_true, y_rul_pred)

 # Asymmetric score
 d = np.array(y_rul_pred) - np.array(y_rul_true)
 asymmetric_score = np.sum(np.where(d < 0, np.exp(-d/13) - 1, np.exp(d/10) - 1))

 # Percentage within bounds
 relative_error = np.abs(d) / np.array(y_rul_true)
 within_10_pct = np.mean(relative_error <= 0.10) * 100
 within_15_pct = np.mean(relative_error <= 0.15) * 100
 within_20_pct = np.mean(relative_error <= 0.20) * 100

 metrics = {
 'fault_classification': fault_report,
 'rul_metrics': {
 'RMSE': rul_rmse,
 'MAE': rul_mae,
 'R2': rul_r2,
 'Asymmetric_Score': asymmetric_score,
 'Within_10%': within_10_pct,
 'Within_15%': within_15_pct,
 'Within_20%': within_20_pct
 }
 }

 return metrics


def generate_results_tables(metrics):
 """Generate formatted results tables."""

 # Fault prediction table
 fault_df = pd.DataFrame(metrics['fault_classification']).T
 fault_df = fault_df.round(4)

 # RUL metrics table
 rul_df = pd.DataFrame([metrics['rul_metrics']])
 rul_df = rul_df.round(4)

 print("=" * 60)
 print("FAULT PREDICTION RESULTS")
 print("=" * 60)
 print(fault_df.to_string())

 print("\n" + "=" * 60)
 print("RUL ESTIMATION RESULTS")
 print("=" * 60)
 print(rul_df.to_string())

 return fault_df, rul_df


# Example usage
if __name__ == "__main__":
 # Generate example predictions for demonstration
 np.random.seed(42)
 n_test = 500

 # Simulated predictions
 y_fault_true = np.random.randint(0, 4, n_test)
 y_fault_pred = y_fault_true.copy()
 # Add some errors
 error_idx = np.random.choice(n_test, size=int(n_test * 0.08), replace=False)
 y_fault_pred[error_idx] = np.random.randint(0, 4, len(error_idx))

 y_rul_true = np.random.uniform(50, 500, n_test)
 y_rul_pred = y_rul_true + np.random.normal(0, 30, n_test)
 y_rul_pred = np.clip(y_rul_pred, 0, None)

 # Class names
 class_names = ['Normal', 'Minor Degradation', 'Severe Degradation', 'Imminent Failure']

 # Sensor names
 sensor_names = [
 'Vib-X1', 'Vib-X2', 'Vib-X3', 'Vib-Y1', 'Vib-Y2', 'Vib-Y3',
 'Vib-Z1', 'Vib-Z2', 'Vib-Z3', 'Temp-Eng', 'Temp-GB', 'Temp-Hyd',
 'Press-H1', 'Press-H2', 'Press-Oil', 'RPM', 'Load', 'Fuel'
 ]

 # Generate attention weights (example)
 temporal_weights = np.random.dirichlet(np.ones(256))
 channel_weights = np.random.dirichlet(np.ones(18))

 # Visualizations
 plot_confusion_matrix(y_fault_true, y_fault_pred, class_names)
 plot_rul_predictions(y_rul_true, y_rul_pred)
 plot_attention_weights(temporal_weights, channel_weights, sensor_names)

 # Compute and display metrics
 metrics = compute_comprehensive_metrics(y_fault_true, y_fault_pred, y_rul_true, y_rul_pred)
 fault_df, rul_df = generate_results_tables(metrics)
